# Global and local neuronal coding of tactile information in the barrel cortex

**DOI:** 10.3389/fnins.2023.1291864

**Published:** 2024-01-05

**Authors:** Hariom Sharma, Rony Azouz

**Affiliations:** Department of Physiology and Cell Biology, Zlotowski Center for Neuroscience, Ben-Gurion University of the Negev, Be'er Sheva, Southern District, Israel

**Keywords:** somatosensory system, whiskers, neuronal synchronization, textures, cortex, sensory processing

## Abstract

During tactile sensation in rodents, the whisker movements across surfaces give rise to intricate whisker motions that encompass discrete and transient stick–slip events, effectively conveying valuable information regarding surface properties. These surface characteristics are transformed into cortical neuronal responses. This study examined the coding strategies underlying these transformations in rat whiskers. We found that changes in surface coarseness modified the number and magnitude of stick–slip events, which in turn both modulated properties of neuronal responses. Global changes in the number of stick–slip events primarily affected neuronal discharge rates and the degree of neuronal synchronization. In contrast, local changes in the magnitude of stick–slip events affected the transformation of these kinematic and kinetic characteristics into neuronal discharges. Most cortical neurons exhibited surface coarseness selectivity through global and local stick–slip event properties. However, this selectivity varied across coding strategies in the same neurons, given that each coding strategy reflected different aspects of changes in whisker-surface interactions. The degree of spatial similarity in surface coarseness preference in adjacently recorded neurons differed among these coding strategies. Adjacently recorded neurons exhibited the same surface coarseness preference in their firing rates but not through other coding strategies. Through these results, we were able to show that local stick–slip event properties contribute to texture discrimination, complementing and surpassing global coding in this context. These findings suggest that the representation of surface coarseness in the cortex may rely on concurrent coding strategies that integrate tactile information across different spatiotemporal scales.

## Highlights

Our sensorimotor system processes enormous amounts of information when interacting with the world. We can construct an internal representation of the environment using these sensory inputs, enabling us to interact with a complex, changing environment accurately. The present results suggest that this process may be accomplished through the ability of neurons to convey multiple tactile parameters through coexisting coding strategies. Notably, different modes of sensory transmission revealed preferential selectivity for various stimulus features. These multi-layered coding schemes enable spike trains to convey information regarding a stimulus through multiple complementary channels, each corresponding to a different aspect of the sensory world and its variations.

## Introduction

Using their whiskers, rats can detect and discern various tactile features of their environment (Brecht et al., 1997; Kleinfeld et al., 2006), including the shape and position of objects (Brecht et al., 1997; Harvey et al., 2001; Mehta et al., 2007; Knutsen and Ahissar, 2009; Kleinfeld and Deschenes, 2011), the width of apertures and gaps (Krupa et al., 2001), and surface texture (Carvell and Simons, 1990; Diamond et al., 2008b; Lottem and Azouz, 2009; Diamond, 2010; Jadhav and Feldman, 2010; Morita et al., 2011; Kuruppath et al., 2014). The active and receptive interactions between the whiskers, given their specific properties (Hartmann et al., 2003; Towal et al., 2011; Hires et al., 2013), and the environment result in frictional movement and induce whisker bending, vibrations, and brief, discrete micromotions referred to as stick–slip events (SSEs) (Diamond et al., 2008a; Wolfe et al., 2008; Lottem and Azouz, 2009; O'Connor et al., 2010; Zuo et al., 2011; Chen et al., 2015; Campagner et al., 2018). The role of the somatosensory system is to decode this information in a manner that enables the accurate determination of the sensed object’s location, shape, and contours.

Several models have been proposed for the neuronal encoding of surface coarseness in the whisker somatosensory system. In one model, the representation of surface coarseness results from the temporal integration of whisker vibration signals within a relatively extended time range. Specifically, this representation is related to the mean speed of surface-induced whisker vibrations (Arabzadeh et al., 2005, 2006; von Heimendahl et al., 2007) and is encoded in the mean firing rate of vibrissal somatosensory cortex (vS1) neurons (Arabzadeh et al., 2003; von Heimendahl et al., 2007; Wolfe et al., 2008; Jadhav et al., 2009). This tactile transformation is termed *global coding*. However, given that SSEs represent significant determinants of overall mean whisker speed, they may still serve as the primary textural cue as they generate most vS1 spikes (Arabzadeh et al., 2005; Lottem and Azouz, 2008; Wolfe et al., 2008; Lottem and Azouz, 2009).

Another plausible coding strategy relies on precise spike timing through the spatiotemporal coordination and synchronization of neuronal assemblies. This synchronization enables neuronal ensembles to encode specific stimulus features better and may serve as an efficient and flexible coding mechanism for sensory and cognitive processing (Gray and Singer, 1989; Softky and Koch, 1993; Dan et al., 1998; Gray, 1999; Azouz and Gray, 2000; Azouz and Gray, 2003; Brette, 2012; Harris and Gordon, 2015). Over the last several years, it has been shown that neuronal synchrony is prevalent in the barrel cortex and thalamus of anesthetized and awake rodents (Zhang and Alloway, 2004; Zhang and Alloway, 2006; Temereanca et al., 2008; Reyes-Puerta et al., 2015; Isbister et al., 2021). This synchrony is present in both thalamic spike timing and membrane potentials in cortical neurons, which were shown to be highly correlated during active touch, thus pointing to a specific synchronization of functional subnetworks (Ferezou et al., 2007; Poulet and Petersen, 2008; Crochet et al., 2011). Our recent study provides strong evidence that the synchronization of barrel cortical neurons is primarily driven by external sensory stimuli (Sharma and Azouz, 2022). Consequently, SSE’s kinetic and kinematic characteristics significantly influence the level of synchrony observed among these cortical neurons.

Finally, recent findings indicated that due to the unique properties of whiskers (Hartmann et al., 2003; Towal et al., 2011; Hires et al., 2013) and their interactions with the environment, they undergo frictional movements resulting in whisker bends, vibrations, and the occurrence of brief, high-velocity, high-acceleration micromotions known as stick–slip events (SSE) (Diamond et al., 2008a; Wolfe et al., 2008; Lottem and Azouz, 2009; O'Connor et al., 2010; Zuo et al., 2011; Chen et al., 2015; Campagner et al., 2018). These SSEs are a significant aspect of interactions between whiskers and surfaces. Thus, the SSE hypothesis suggests tactile information is encoded as probabilistic stick–slip movements in whiskers. The kinematic profiles of SSEs carry information related to texture (Ritt et al., 2008; Wolfe et al., 2008), and are effectively processed by neurons along the ascending tactile pathway (Pinto et al., 2000; Jones et al., 2004; Arabzadeh et al., 2005; Jadhav et al., 2009; Jadhav and Feldman, 2010; Stuttgen and Schwarz, 2010; Waiblinger et al., 2013, 2015; Allitt et al., 2017). Hese SSEs trigger low-probability responses in the primary somatosensory cortex (S1) (Simons, 1978; Pinto et al., 2000; Kerr et al., 2007; Stuttgen and Schwarz, 2008; Crochet et al., 2011). These SSE quantity and kinematic patterns change with surface roughness and are reflected in altered spike probabilities in S1 (Jadhav et al., 2009; Isett et al., 2018). This coding mode through SSEs differs notably from previous strategies due to its localized spatiotemporal nature (Schwarz, 2016).

The present study compared various cortical coding strategies for surface coarseness by analyzing whisker vibrations in response to different textures and recording vS1 neural activity in anesthetized rats. Beyond measuring neuronal discharge rates, we identified temporal coding as a crucial aspect of sensory-evoked activity in the barrel cortex. This temporal coding is characterized by the synchronous occurrence of a subset of spikes within the neuronal population, forming a dynamically relevant subnetwork (Palm, 1990; Hebb et al., 1994; Singer, 1999; Lestienne, 2001; Womelsdorf and Fries, 2007; Cohen and Kohn, 2011; Reyes-Puerta et al., 2015; Isbister et al., 2021). Furthermore, our findings reveal that vS1 neurons encode the amplitude of surface-induced SSE through sparse, low-probability, precisely timed spikes during continuous contact with surfaces. This transformation of SSEs into response probabilities and the temporal dynamics of neuronal responses is a robust coding strategy for representing surface coarseness. Our results suggest multiple coding strategies capture the various facets of surfaces and objects.

## Materials and methods

### Animals and surgery

Male and female Sprague–Dawley rats weighing 250–320 grams were administered anesthesia for the experiment. The anesthesia protocol involved the administration of ketamine (100 mg/kg, i.p., Ketaset; Fort Dodge Animal Health, Fort Dodge, IA) and acepromazine maleate (1 mg/kg, i.p, PromAce, Fort Dodge Animal Health). After performing a tracheotomy, a metal cannula (1.5 cm in length) with an outer diameter (o.d.) of 2 mm and an inner diameter (i.d.) of 1.5 mm was inserted into the trachea. Subsequently, the rats were placed in a standard stereotaxic device, and their body temperature was maintained at 37.0 ± 0.1°C using a heating blanket and a rectal thermometer (TC-1000; CWE, Ardmore, PA). Anesthesia was sustained by delivering a mixture of halothane (0.5–1.5%) and air through artificial respiration at 100–115 breaths per minute. End-tidal CO2 levels and heart rate were monitored throughout the procedure to ensure proper anesthesia depth. The anesthesia level was evaluated based on heart rate (250–450 bpm), eyelid reflex, pinch withdrawal, and vibrissal movements. Halothane concentrations were adjusted slightly above where the first noticeable signs of vibrissal movements appeared while maintaining the eyelid reflex.

In some cases, EEG recordings were conducted by inserting two wires beneath the skull at a distance of 10 mm anterocaudally. Based on these measurements, the anesthesia level during the recordings was classified between stages III-2 and III-3 (Friedberg et al., 1999). Following placement of the subjects in a stereotactic apparatus (TSE, Bad Homburg, Germany), a small opening (1–2 mm in diameter) was created above the barrel cortex, precisely centered at 2.5 mm posterior and 5.2 mm lateral to the bregma, and the dura mater was cautiously removed.

We investigated the relationship between microdrive depth and laminar identity in a subset of animals. To determine the corresponding areas, we performed electrolytic lesions using the recording electrodes, applying a direct current (10–30 μA) for 4 s at a depth related to each specific recorded area. Additionally, in select rats, brain tissues underwent cytochrome oxidase histochemistry processing. The animals were transcardially perfused with 2.5% glutaraldehyde and 0.5% paraformaldehyde, followed by 5% sucrose, all in 0.1 M phosphate buffered saline (PBS). The brains of these rats were then transferred to a 30% sucrose post-fixative solution and incubated overnight at 4°C. Subsequently, microtome cryosections (120 μm) were prepared and incubated in a solution of PBS containing 0.0015% cytochrome C (Sigma) and 0.05% diaminobenzidine for 20 to 50 min at 37°C. The reaction was halted by rinsing with PBS. The cytochrome oxidase-stained sections were mounted on gelatin-coated slides, air-dried, and coverslipped. Laminar identification was based on specific recording depths: layers 2/3, 4, 5, and 6 were characterized by depths of 150–550 μm, 550–850 μm, 900–1,400 μm, and 1,400 μm and deeper, respectively.

All experiments were conducted in accordance with appropriate international standards and were approved by the Ben-Gurion University (BGU) Committee for the Ethical Care and Use of Animals in Research (project license: IL-71-11-2016). The BGU animal care and use program is supervised and fully assured by the Israeli Council for Animal Experimentation of the Ministry of Health. It is operated according to Israel’s Animal Welfare Act of 1994 and follows the Guide for Care and Use of Laboratory Animals (NRC 2011). In addition, BGU is approved by the Office of Laboratory Animal Welfare, United States (OWLA) (#A5060-01). Sprague–Dawley rats (250–300 g) were used for all experiments described herein.

### Recording technique

An implanted multi-contact silicone electrode (NeuroNexus, Ann Arbor, Michigan) was carefully inserted into the barrel cortex using a precise stereotactic micromanipulator (TSE-systems, Germany). Throughout the recording process, the signals were amplified (1,000×), digitized (25 kHz), and filtered (0.1–10,000 kHz), after which they were stored for offline spike sorting and analysis. The ME-16 amplifier and MC-Rack software (MEA, Germany) were employed for this purpose. The recorded data was separated into local field potentials (LFP; 1–150 Hz) and isolated single-unit activity (0.5–10 kHz). All the neurons under study exhibited responsiveness to manual stimulation of at least one whisker. To extract and sort the spikes, we utilized the MClust MATLAB-based spike-sorting software developed by A.D. Redish.^1^ The resulting spikes were stored with a temporal resolution of 100 μs, and peri-stimulus time histograms (PSTHs) were subsequently computed (Supplementary Figure S1).

### Whisker stimulation

To simulate different surface textures and study whisker movements during receptive sensing in awake-behaving rats, we covered the face of a rotating cylinder with various grades of sandpaper, each offering a different level of coarseness. The cylinder was rotated against the whiskers of the subject rats, with the whiskers resting upon the cylinder face. This setup aimed to mimic the natural rostral-caudal movement of the whiskers during head motion. The head velocities associated with rat exploration were obtained from previous studies (Lottem and Azouz, 2009; Gugig et al., 2020) and used as a reference for controlling the velocity of the rotating cylinder. A DC motor was employed to drive the 30 mm diameter wheel, maintaining a speed of approximately 147 degrees per second to replicate median head velocity. For this study, we utilized sandpaper surfaces with five different coarseness grades: P120 (125 μ), P220 (68 μ), P400 (35 μ), P600 (25 μ), and P800 (21 μ). These grades were selected based on previous research (Arabzadeh et al., 2005; Hipp et al., 2006; Morita et al., 2011). Whisker displacements were captured using a Mikrotron CoaXPress 4CXP camera, recording at 1,600 frames per second with a resolution of 4 Megapixels. The camera was positioned above the arena, providing an overhead view of the whisker movements. All recorded videos were analyzed using the Janelia whisker tracker software (Clack et al., 2012). To estimate the curvature of the whisker and determine the forces exerted on the whisker follicle (Birdwell et al., 2007; Towal et al., 2011; Quist and Hartmann, 2012), we employed the methodology previously described by Birdwell et al. (2007). We measured curvature at ten locations along the whisker for this analysis and extracted the maximum local curvature from each image.

### Data analysis

**Figure 1:**
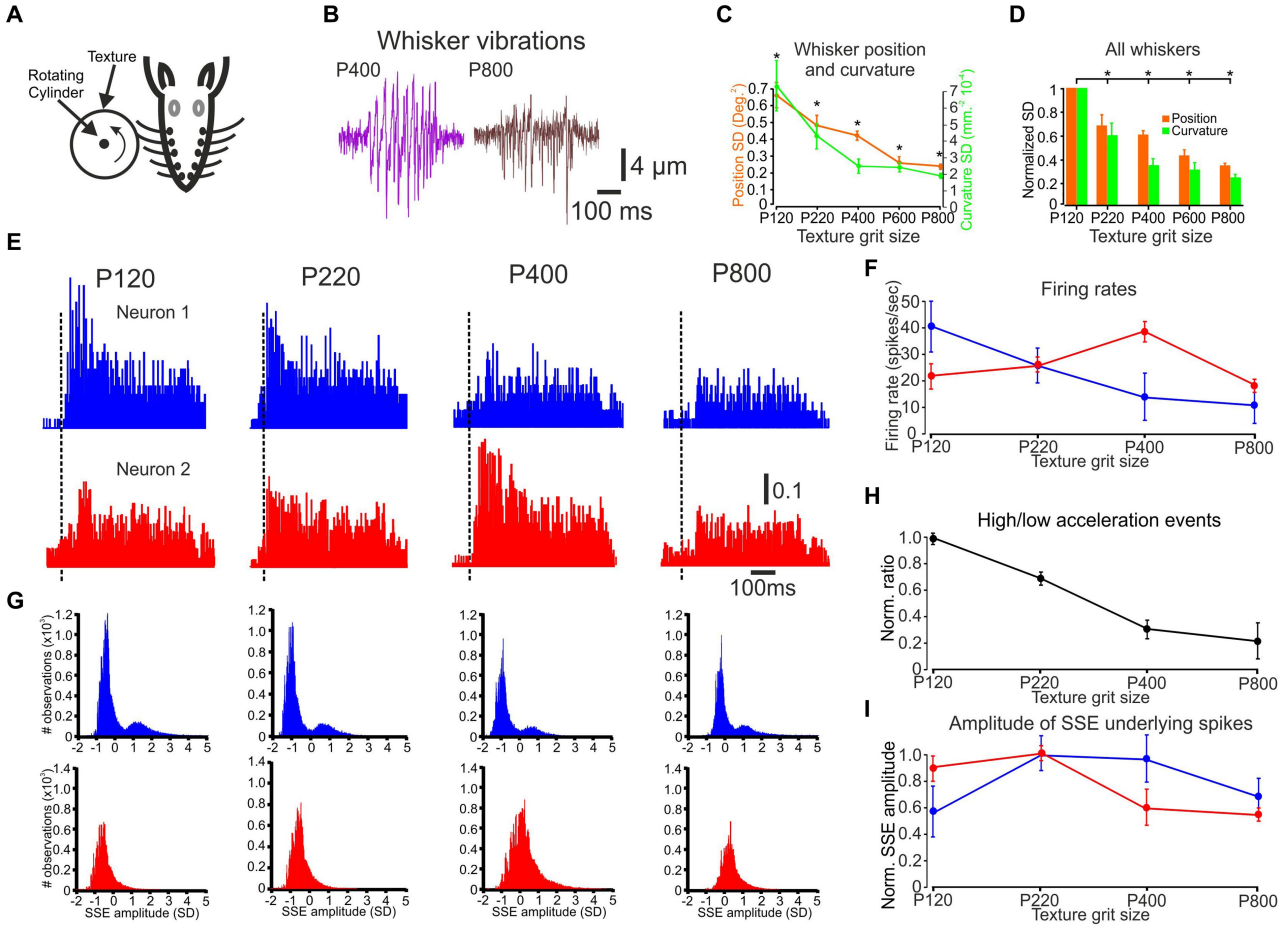
The influence of changes in surface coarseness on whisker vibrations, neuronal discharge rates, and SSE amplitude. **(A)** An overview of the experimental design, demonstrating that whiskers are placed in contact with a rotating cylinder covered with textured sandpaper. **(B)** An example of C3 whisker movements in response to P400 and P800 textures. The vertical line on the whisker vibration scale bar indicates the image measurement and the horizontal line indicates the time in milliseconds. **(C)** The influence of texture coarseness on the SD of whisker vibration position (orange) and curvature (turquoise) for the whiskers in **B**. Asterisks indicate statistically significant differences between the groups (*F* (4, 37,495) = 110.3, *p* = 3×10^−3^). **(D)** The influence of texture coarseness on the SD of whisker vibration position (orange) and curvature (turquoise) for all recorded whiskers. All SD values were normalized to P120. Asterisks indicate statistically significant differences between groups (*F* (4, 19) = 12, p = 3×10^−4^). **(E)** PSTHs of two neurons were recorded simultaneously. The scale bar for the PSTH represents the probability of firing for a 1 msec bin. **(F)** The influence of texture grit size on neuronal firing rates of the neurons in **E**. **(G)** Normalized distribution of all SSEs that resulted in spikes of the neurons in **E**. **(H)** Ratio of the number of high-to low-acceleration events as a function of surface coarseness. Data shown in **H** are from all recorded whiskers; error bars represent the standard error calculated across all whiskers. **(I)** The influence of texture grit size on mean SSE resulted in spikes of the neurons in **E**.

We established a trial structure to examine the influence of surface coarseness on whisker motion and resulting cortical neuronal responses. The cylinder rotated for 500 ms for each texture and remained still for 1,500 ms. This procedure was repeated 75–150 times. We then aligned the whisker responses and the corresponding neuronal responses to the beginning of cylinder movement to generate PSTHs (Figures 1E,F).

The electrophysiological data was sampled at a frequency of 25KHz. The resulting spikes were stored with a temporal resolution of 100 μs. In parallel, whisker movements were recorded in a video format at a rate of 1,600 frames per second. To establish the correlations between these two data streams, the two signals were aligned. This was primarily accomplished by subsampling the spike timing information at 1 ms.

The significance of the differences between measured parameters was evaluated using a one-way analysis of variance (ANOVA). When significant differences were indicated in the F ratio test (*p* < 0.05), Tukey’s multiple comparisons method was used to determine those pairs of measured parameters that differed significantly from each other within a group of parameters (*P* < 0.01). The results are presented as the mean ± standard deviation (SD). Error bars in all the figures indicate the SD unless otherwise noted. To avoid cluttering some of these graphs, single-sided error bars were used.

### Receiver operating characteristics analysis

We used signal detection theory (receiver operating characteristics [ROC] analysis; Green and Swets, 1974) to compute the probability that an ideal observer could accurately determine the differences among the different textures based on neuronal activity. For each measured texture pair neuronal responses, an ROC curve was constructed as a two-dimensional plot of hit probability (y-axis) and a false alarm (x-axis) probability. Green and Swets (1974) demonstrated that the area under the ROC curve (AUC) corresponds to the performance expected of an ideal observer in a two-alternative, forced-choice paradigm, such as the one used in the present analysis. The ROC curve was calculated for a single neuron’s firing rate as a texture function. We then averaged all AUC values of all neurons and all texture pairs.

To transform raw data into a measure of discriminability, we analyzed the distributions of neuronal firing rates across trials. The firing rate (*Fr*) in trial *k* is the spike count = $Nsp_{k}$ divided by *T*, trial duration in ms.

$$Fr=\frac{Nsp_{k}}{T}$$

The length *T* for the texture signal was set to *T* = 500 ms.

To assess the significance level of the AUC values we got from each neuron for all texture comparisons, we shuffled the firing rates of all trials among the various textures. We then calculated the ROC curves and AUC values for the shuffled data. We then averaged all AUC values of all neurons and all texture pairs. This process was repeated 500 times. The significance level, set at mean + 2SD (95%), equated to AUC = 0.53.

**Figure 2:**
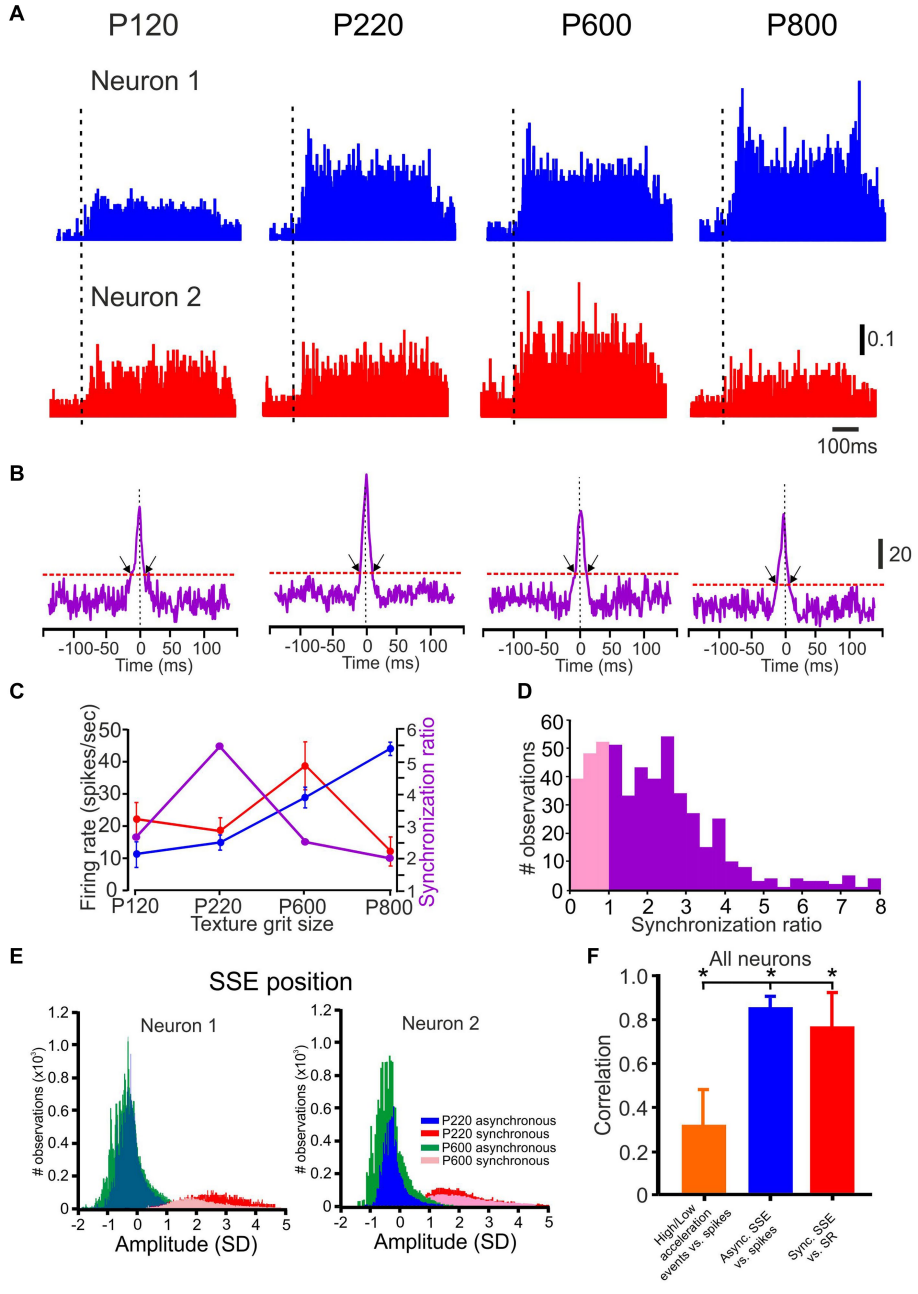
Neuronal synchronization as a function of surface coarseness. **(A,B)** PSTHs and CCHs correspond to P120, P220, P600, and P800 textures. The vertical scale bar for PSTH shows the spike probability. In the PSTHs, the dashed vertical line indicates the starting point of the stimuli. The vertical scale bar of the CCHs shows the number of spikes at zero time lag. The black arrows indicate the time window of significant synchronous spikes. The scale bar for the PSTH represents the probability of firing for a 1 msec bin. **(C)** The influence of surface coarseness on neuronal firing rates and neuronal synchronization for the neurons in **A**. **(D)** Distribution of SR values in all neurons. The pink distribution shows the SR values below one. **(E)** Normalized distribution of SSE amplitudes for the neurons in A-B resulted in asynchronous (blue and turquoise) and synchronous (red and pink) spikes for two textures. **(F)** The correlation between the low-to high-acceleration events ratio and firing rates (orange), between firing rates and the number of asynchronous SSE (blue), and between SR values (degree of synchronization) and the number of synchronous SSE (red) in all neurons.

### Texture selectivity

A neuron responding to several textures shows a higher or lower firing rate for a particular texture, and this neuronal property is referred to as texture selectivity (Garion et al., 2014). An additional criterion for texture selectivity implemented was determining whether a specific texture had a significantly higher or lower firing rate (or any other parameter) than all other textures.

To calculate the texture selectivity of cortical neurons, we used the Selectivity Index (SI).

𝑆𝐼 = 𝑀𝑎𝑥(𝑃𝑖) − $\left(\bar{pj}\right)$ /𝑀𝑎𝑥(𝑃𝑖)

Where *P* is the firing rates; *i* = preferred texture; *j* = all texture excluding the preferred texture; *Max*(*Pi*) = maximal firing rate; $\left(\bar{pj}\right)$ = the average firing rates across all textures.

To quantify the statistical significance of texture selectivity, we first calculated the SI for several textures using the SI formula outlined above. Second, for each neuron, we have *n* × 75 trials, where *n* represents the number of textures, and 75 signifies the number of trials conducted for each texture. We randomly shuffle all trials across different textures to compute the SI. We iterate this process 500 times, calculating the average surrogate SI and SD afterward. We calculated the ‘mean + 3SD’ from this 500 SI data distribution. If the original SI surpassed the surrogate SI (mean + 3SD), this provided confirmation that the texture selectivity was not merely a product of chance.

### Surface coarseness impact on neuronal responses

**Figure 3:**
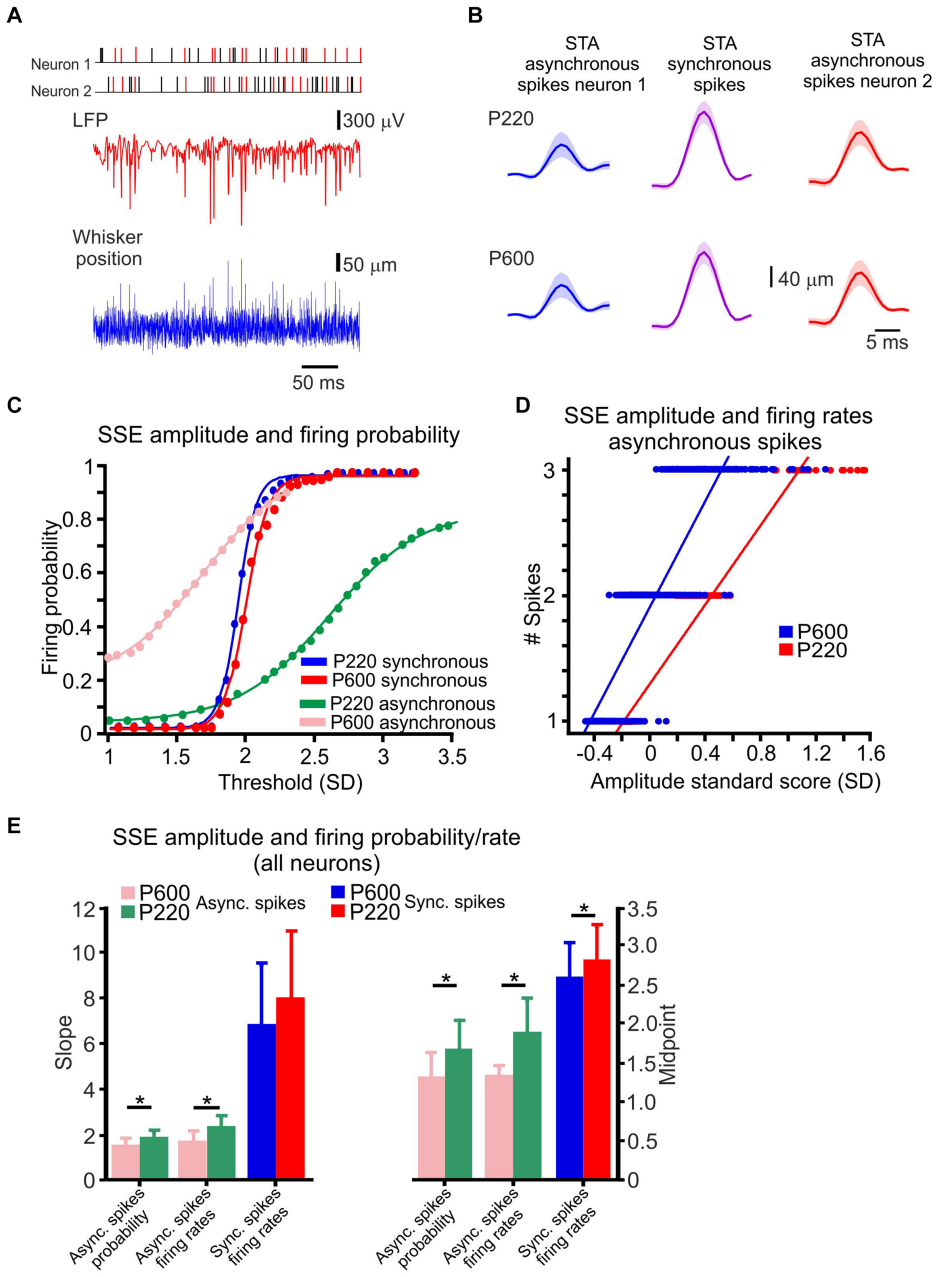
Cortical neuronal responses to textures are composed of intermingled synchronous and asynchronous spikes. **(A)** An example of whisker motion, LFP, and spike discharge in two neurons in response to the P220 texture. The spikes marked in red are synchronous. **(B)** STA of whisker motion, synchronous (middle panels), and asynchronous spikes (left and right). **(C)** The relationship between detection threshold and firing probability for the neurons in **B** and Figure 2, in asynchronous and (pink and green) and synchronous (red and blue) spikes for the two textures. This relationship fits a sigmoidal function. **(D)** The relationship between SSE amplitude and firing rates for asynchronous spikes for one of the neurons in **B** for P220 (red) and P600 (blue) textures. Lines indicate the results of a linear regression analysis. **(E)** Population statistics for asynchronous and synchronized spikes for the two textures. The slope of the fit line for the firing rates and probability for asynchronous spikes exhibited a significant change as a function of surface coarseness. In contrast, the slope of synchronous spikes did not change. The shift in the fits for both types of spikes exhibited a significant change as a function of surface coarseness. Asterisks indicate significant differences (asynchronous spikes probability slope: *t* (126) = 6.12, *p* = 0.014231; asynchronous spikes firing rates slope: *t* (126) = 10.544, *p* = 0.0015275; asynchronous spikes probability midpoint: *t* (126) = 25.626, *p* = 1.5901e^−06^; asynchronous spikes firing rates midpoint: *t* (126) = 15.994, *p* = 0.00011269; synchronous spikes firing rates midpoint: *t* (126) = 9.48, *p* = 0.001348).

**Figure 4:**
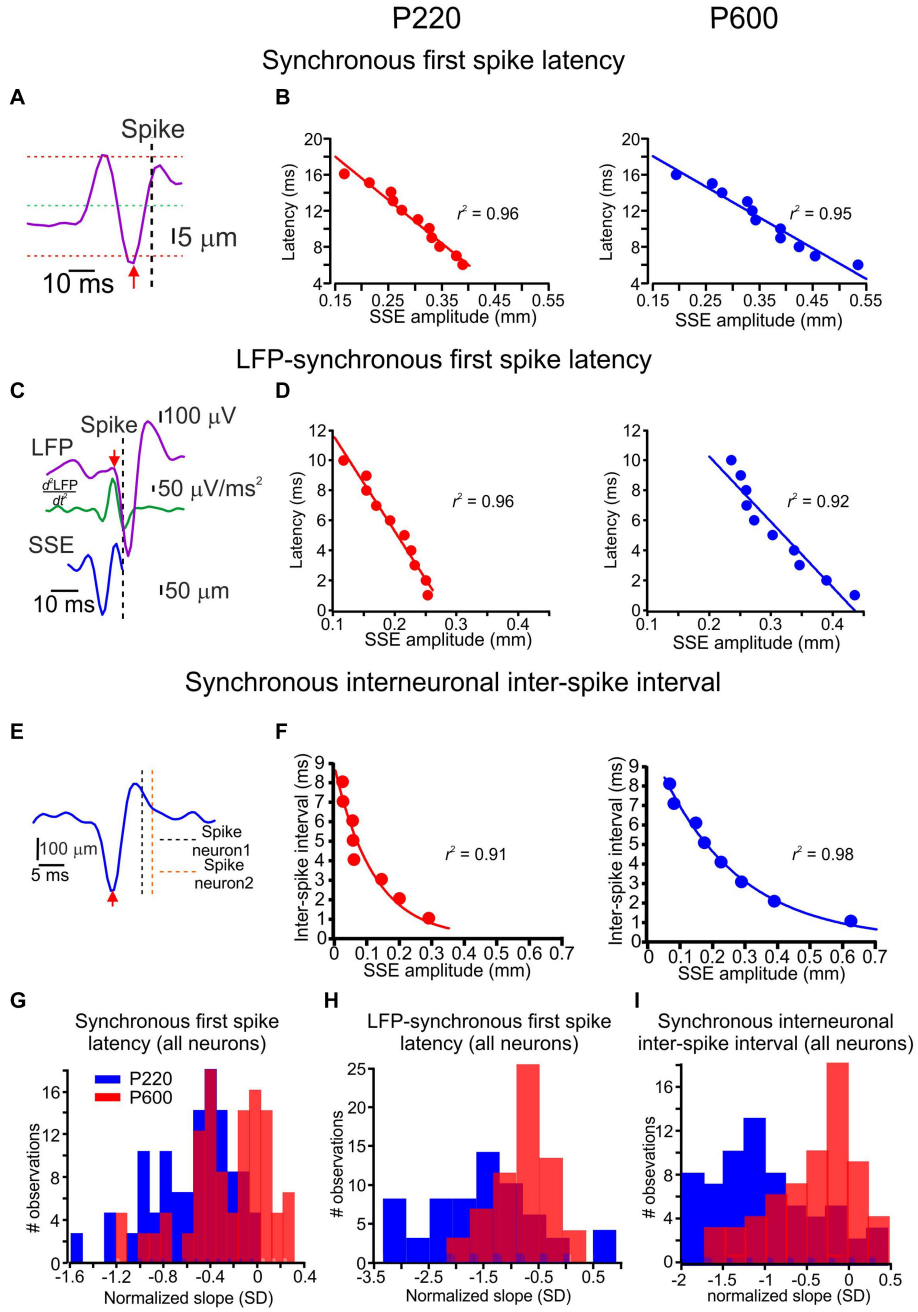
Coding of surface coarseness through local features. **(A)** Spike latency from its underlying single SSE (red arrow to vertical dashed line) for synchronous spikes. **(B)** The relationship between SSE amplitude and spike latency in synchronous spikes for the P220 and P600 textures (left and right panels, respectively). Each point in the graphs represents the mean. The line is the linear regression fit of the data. **(C)** Spike latency from its underlying LFP (red arrow to vertical dashed line) for synchronous spikes from a single SSE. The LFP commencement was determined by the second derivative of the LFP (turquoise traces; see Methods). **(D)** The relationship between SSE amplitude and LFP-spike latency in synchronous spikes for P220 and P600 textures (left and right panels, respectively). The line is the linear regression fit of the data. **(E)** Interneuronal ISI (vertical dashed lines) from its underlying single SSE (red arrow) for synchronous spikes. **(F)** The relationship between SSE amplitude and interneuronal ISI for synchronous spikes. The line is the exponential decay fit of the data. **(G)** Distribution of the normalized linear regression fit slopes for all neurons’ relationships between SSE amplitude and spike latency for P220 (red) and P600 (blue). **(H)** Distribution of the normalized linear regression fit slopes for all neurons’ relationships between SSE amplitude and LFP-spike latency for P220 (red) and P600 (blue). **(I)** Distribution of the normalized linear regression fit slopes for all neurons’ relationships between SSE amplitude and synchronous spike interneuronal ISI for P220 (red) and P600 (blue).

**Figure 5:**
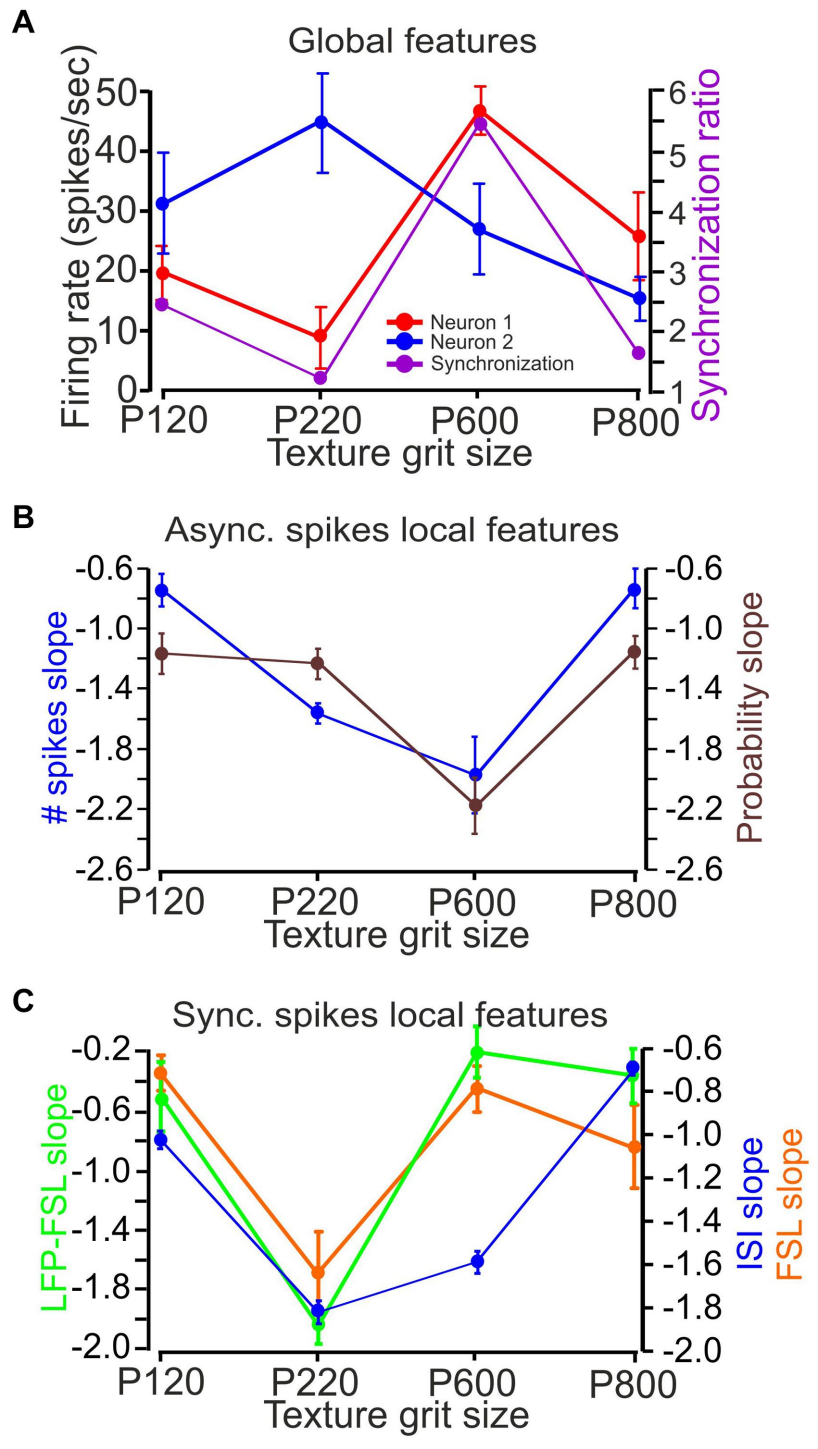
The influence of surface coarseness on firing rates, synchrony, and local features. **(A)** The impact of surface coarseness (for neurons from Figures 3 4) on neuronal firing rates in paired recordings (red and blue) and neuronal synchronization (purple). **(B)** Asynchronous spike local features [number of spikes (blue) and probability (black)]. **(C)** Synchronous spike local features [first spike latency (orange), LFP-first spike latency (turquoise), and ISI (blue)].

**Figure 6:**
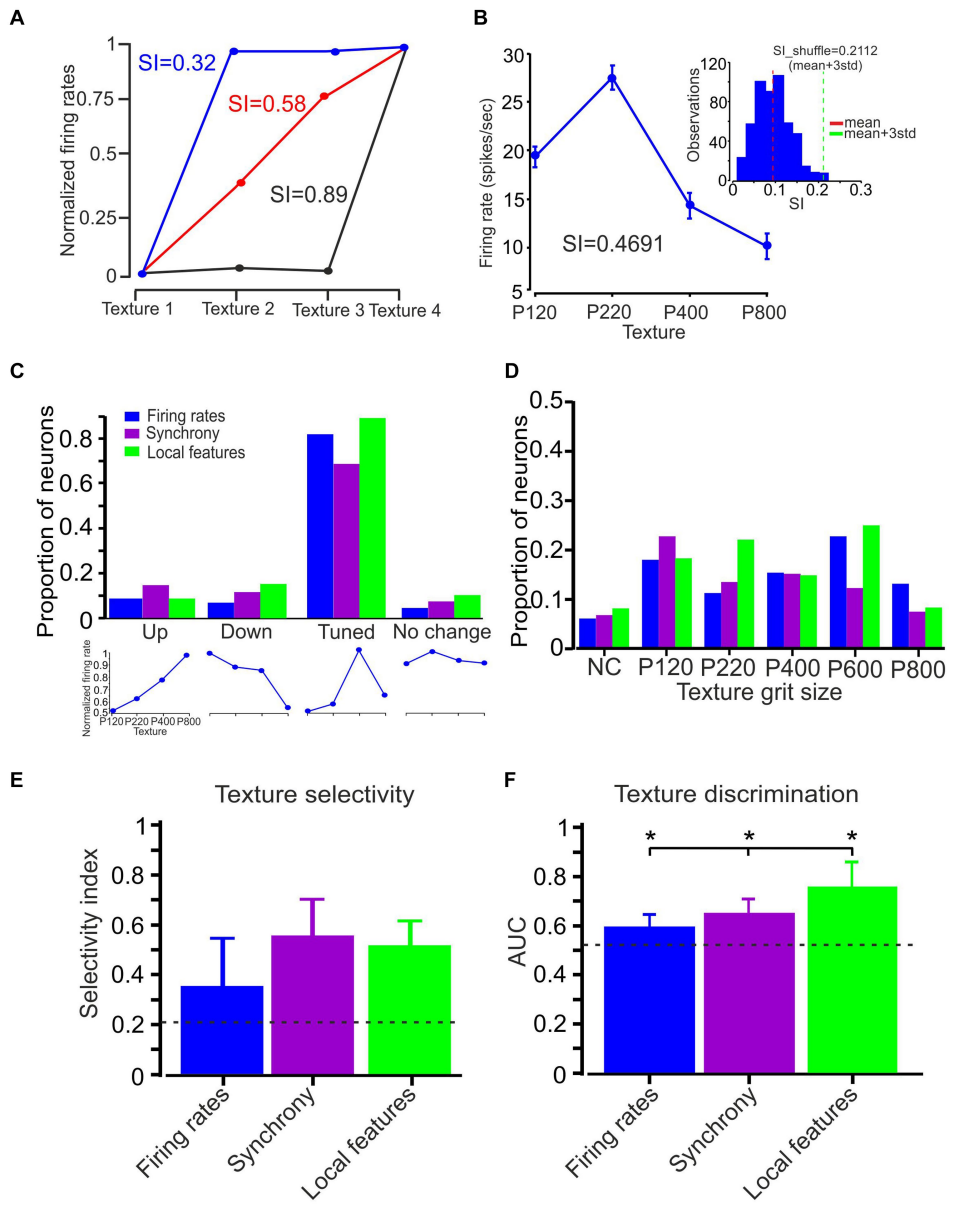
Neuronal selectivity and discrimination. **(A)** Quantification of texture selectivity. The plot compares the normalized firing rate for the different textures. Three different numerical values present the SI corresponding to these three conditions. **(B)** Average firing rate (75 trials) associated with textures P120, P220, P400, and P800.The SI value was 0.4751 (calculated using the formula in the Methods section). The inset shows the statistical significance of texture selectivity. The histogram shows the distribution of 500 SI values. The red and turquoise vertical lines represent the mean and mean + 3SD of the SI data distribution, respectively. The mean + 3SD of the SI data distribution was 0.21. **(C)** Four different groups of neurons based on the relationship between surface coarseness and neuronal responses (up, down, tuned, and no change; lower panels; see text). Neuronal firing rates (blue), synchrony (purple), and local features (turquoise) show that most of the neurons in the different coding strategies are tuned to a specific texture. **(D)** The coarseness preferring neuronal population was further subdivided according to their texture coarseness preference. **(E)** Mean selectivity index values for neuronal firing rates, synchronization, and local features. The error bars represent the SD of SI. Asterisks indicate significant differences. **(F)** The mean AUC values for neuronal firing rates (blue), synchronization (purple), and local features (turquoise) across all textures in all neurons. Error bars represent the SD. Asterisks indicate significant differences.

Upon plotting the neuronal response characteristics corresponding to various textures, we discern a complex and interconnected relationship between these parameters, as depicted in Figures 1–6. To quantify these complex relationships, we divided the neural responses as a function of surface coarseness into four categories (Figure 6C lower panels):

1. Up - neurons presenting a significant monotonic increase.
2. Down - neurons presenting a significant monotonic decrease.
3. Tuned - neurons exhibiting a preference for a specific texture (reduction and increase).
4. No change - neurons that did not show any significant changes.

In order to categorize the diverse dependencies observed, we established specific empirical rules. These rules were strategically designed to classify these groups based on visually discernible characteristics distinctly. They were set to be both minimal and comprehensive enough to divide the dependencies into their respective visually inspected groups accurately. We discovered that, for categories 1 and 2, when at least 3 out of 4 neuronal responses to different textures (4 textures) displayed consistent and statistically significant ascending (upward) or descending (downward) trends in various aspects of their neuronal responses, it corresponded to the visually inspected dependencies.

### Spatial selectivity similarity

To examine the degree of spatial clustering of texture selectivity of neurons recorded from the same tetrode site (<150 μm). We devised a similarity measure between adjacent neurons, termed *Spatial Selectivity Similarity*, calculated as the number of neurons selective to the same texture divided by the total number of neurons in a particular cluster. A cluster refers to a group of neurons that have been recorded from a single tetrode site.

$$\mathrm{Similarity value}=\frac{N\;sameselectivityincluster\;}{N\;total}$$

To gauge the significance level, we meticulously computed a comprehensive array of potential scenarios encompassing various cluster sizes, explicitly focusing on clusters ranging from 3 to 5 neurons. This analysis was conducted across datasets involving 4 and 5 textures, enabling a thorough examination of the diverse combinations and their significance within the study. We calculated the expected probability for all possible variations in the different conditions.

$$\begin{array}{l} Theexpectedprobability=\\ \;\;\overset{texture\;n}{\underset{i=texture1\;}{\sum }}\frac{\;N_{i}}{\sum N_{texture}}*\frac{N\;sameselectivityincluster}{N\;total} \end{array}$$

**Figure 7:**
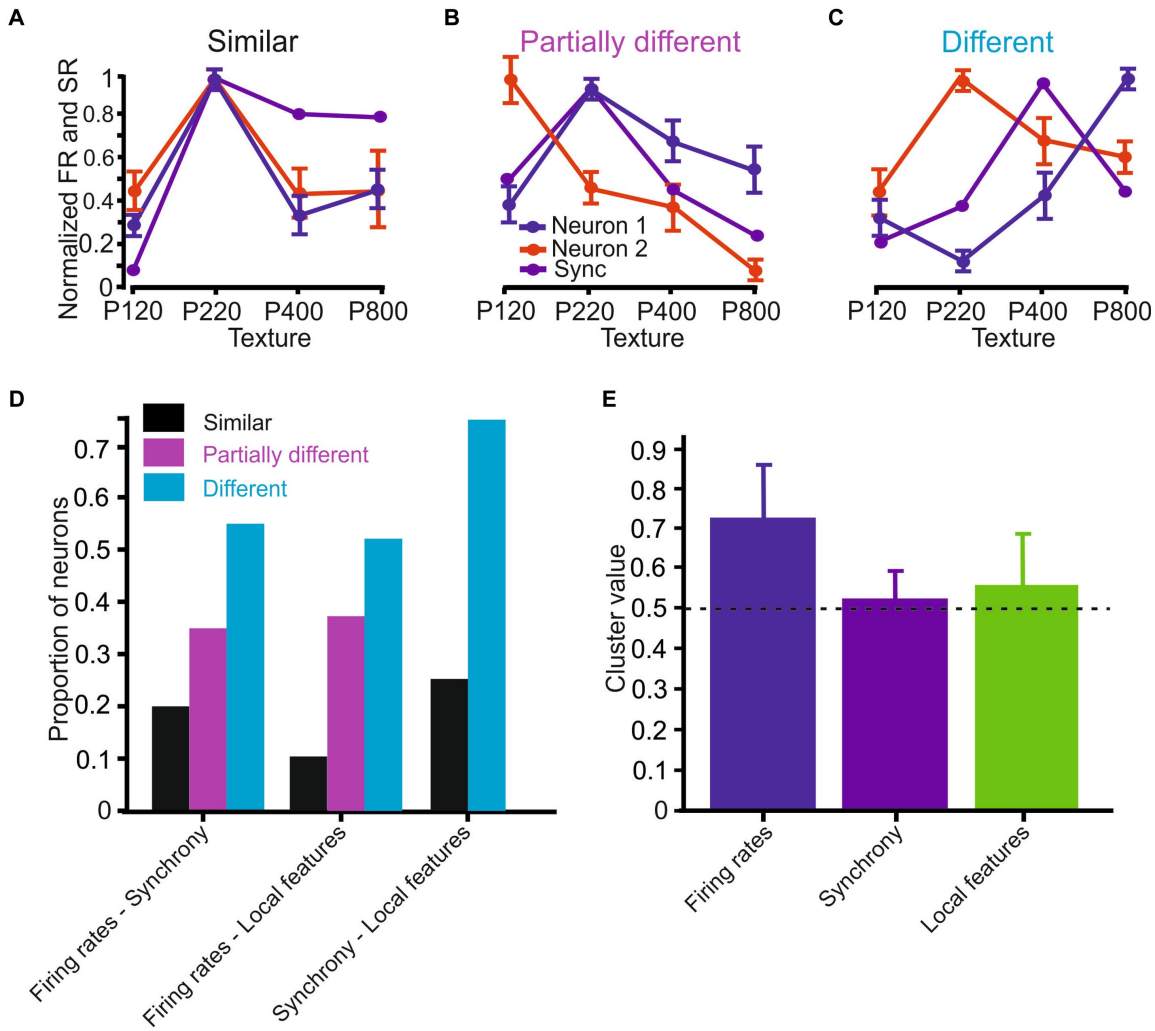
Surface coarseness preferences in the different coding strategies. **(A–C)** Neuronal response similarity categories: **(A)** Similar – all coding strategies show the same preference. **(B)** Partially similar - some coding strategies show the same preference. **(C)** Different – none of the coding strategies show the same preference. **(D)** Proportions of neurons in the different categories. **(E)** Mean spatial clustering values in the various coding strategies. The dashed horizontal line indicates the significance level (see text).

We found that for three neurons, the respective significance levels for 4 and 5 textures were 0.49 and 0.56. Figure 7E shows the significance level for three neurons and four textures.

### Quantification of temporal synchronization

To compute the cross-correlation of a spike train, we used the method described previously by Maldonado et al. (2000). We represented the spike train of each neuron as a binary time series with 1 ms resolution such that:

$$c_{j}^{i}\left(t\right)=\{\begin{array}{c} 1,if\;on\;trial\;i,neuron\;j\;fired\\ anactionpotentionalduring\;\\ the\;t^{th}\;ms;\\ 0,otherwise \end{array}$$

We then computed the cross-correlogram histogram (CCH) that represents how two neurons tend to fire in conjunction with one another:

CCH( τ )= $\overset{M}{\underset{i=1}{\sum }}\overset{N}{\underset{t=1}{\sum }}c_{1}^{i}$ (t) $c_{2}^{i}\left(t+\tau \right)$

Where M represents the number of trials, N is the number of bins in the trial, $\;c_{1}^{i}$ and $c_{2}^{i}$ are the spike trains of cells 1 and 2 on trial *i*, and τ is time lag.

To quantify the temporal synchronization of the correlated firing rate, which occurred within ±10 ms of the zero time lag, we used the significance ratio (SR). SR was computed as the ratio of two integral values: a peak value (*P*), representing the magnitude of the spike correlation, which is calculated by taking the sum of the bins in the central 20 ms of the cross-correlogram that exceeds the 95% confidence limit, and a variance value (V) representing the expected occurrence of coincident spikes, which is computed from the sum of the central 20 ms in each histogram lying between the 99% confidence limit and the mean value of the correlogram. We computed the SR as follows:

SR = P/V

where

$$P=\overset{i=10}{\underset{i=-10}{\sum }}\left(pbin_{i}\right)+\overset{j=10}{\underset{j=-10}{\sum }}\left(nbin_{j}\right)$$

and

$$pbin_{i}=\left(bin_{i}-\left(X+2\sigma \right)\right)\;\;when\;bin_{i}>X+2\sigma$$

$$nbin_{i}=\left(\left(X-2\sigma \right)-bin_{j}\right)\;\;when\;bin_{j}<X-2\sigma$$

and where

$$V=\overset{i=10}{\underset{i=-10}{\sum }}\left(pxbin_{i}\right)+\overset{j=10}{\underset{j=-10}{\sum }}\left(nxbin_{j}\right)$$

$$pxbin_{i}=2\sigma \;when\;bin_{i}\geq X-2\sigma$$

$$pxbin_{i}=bin_{i}-X\;when\;X+2\sigma$$

$$nxbin_{j}=2\sigma \;when\;bin_{j}\leq X-2\sigma$$

$$nxbin_{j}=X-bin_{j}\;when\;X>bin_{j}>X-2\sigma$$

Where *X* is the mean value of all bins in the cross-correlogram, and σ is the standard deviation of all the bins in the corresponding control correlogram.

Each CCH was computed from the cross-correlation between each trial of two neurons and the corresponding cumulative correlogram was calculated. The SR value was computed for each CCH. For each experimental trial, we computed an equivalent pseudorandom trial (i.e., a random trial taken from 75 trials) and a shuffled trial obtained by randomly shuffling 75 trials. This method, using surrogate data, conserves the PSTH shapes of the neuron pairs.

To further examine the influence of the temporal pattern of spikes and the significance of the temporal locking of spikes to the stimulus, we also computed an equivalent pseudorandom trial in which we shuffled the inter-spike intervals within each trial. We created surrogate data that included an equal number of spikes and the same ISI distribution in each trial. *This procedure shuffled spike timing and eliminated temporal locking to the SSE while keeping the number of spikes and the ISI distribution constant.* These two simulations were repeated 500 times for each CCH on each trial. This yielded 500 control SR values (pseudorandom and inter-trial shuffled) for each experimental CCH. We assigned a confidence limit for statistical significance by choosing the SR values in the control distribution that were >99% of the values. An SR ratio >1 was considered significant. The designation “control correlogram” has been assigned to these two methodologies used to generate surrogate data for analysis.

### Synchrony-based selection

Initially, we focused on identifying neurons that displayed noticeable synchronized activity in their spike trains. Once identified, we determined the time window for significant correlation based on the central peak observed in the cross-correlogram (CCH) analysis. Within this selected group of neurons, we specifically examined the interneuronal inter-spike intervals (ISIs) between pairs of neurons. If the ISIs between two neurons fell below the chosen time window, we classified the corresponding spikes as synchronized spikes, indicating their simultaneous occurrence. Conversely, if the interneuronal ISIs exceeded the selected time window, we categorized the spikes as asynchronous spikes, indicating their non-simultaneous occurrence.

### Spike-triggered average calculations

To compute the Spike-triggered average (STA) kernel, the signal related to whisker position, velocity, acceleration, or curvature was averaged over a specific time window, with a kernel width of 20 ms, preceding every spike event (Schwartz et al., 2006). By using various types of spikes (synchronous and asynchronous spikes, see above) as triggers and selecting different signal components, we derived different STA variants, namely STAsync and STAasync. When analyzing the LFPs, we averaged the LFP signal over the 20 ms preceding each spike and the 7 ms following it.

### Detection and quantification of SSEs underlying spikes

To examine the relationship between SSE characteristics and the different response properties, we first defined SSE as a significant change in whisker movement prior to each spike. SSE was detected preceding each spike by identifying a peak value in whisker movement that crossed the threshold within 20 ms preceding each spike. The peak value was defined as the time of the SSE event. The threshold used in the current study was the mean ± 3 SD (see Figure 4; Supplementary Figure S2; different threshold levels did not change our results; Sharma and Azouz, 2022). The amplitude of SSE preceding each spike was calculated by measuring the peak-to-peak amplitude within ±10 ms of the largest peak that crossed the threshold. To further verify this method, we calculated the SSE-spike latency. Our findings revealed minimal latencies at L4 (mean ± SD: 6.8 ± 0.8 ms, *n* = 72 neurons). At L2/3, latencies were longer (mean ± SD: 10.3 ± 0.9 ms, *n* = 82), while L5 and L6 displayed even greater latencies (mean ± SD: 9.5 ± 1.6 ms, *n* = 69; 13.6 ± 0.9 ms, *n* = 47, respectively; Narayanan et al., 2017; Sharma and Azouz, 2022).

### SSE-dependent firing rates

Once we identified SSEs that led to spike occurrences, we quantified the number of spikes that occurred in the aftermath of each SSE. Specifically, we investigated how the amplitude of SSEs influenced the spike count within a subsequent 30 ms time window, explicitly focusing on asynchronous spikes (Figure 4E).

### SSE-dependent firing probability

To investigate how SSE amplitude affects the probability of spike discharge, we implemented a dynamic threshold to detect whisker vibration events. We determined a specific threshold for each of these events and examined the number of spikes occurring within a subsequent 30 ms window. By calculating the ratio of the number of spikes to the number of events, we obtained the probability of spike discharge for each threshold value (Figure 4D).

### Stimulus-dependent LFP commencement

For every spike triggered by sensory stimuli, we determined the corresponding LFP. To identify the onset of each LFP, we computed the second derivative of the LFP signal. By analyzing the inflection points obtained from the second derivative (indicated by red arrows in Figure 5B, inset), we calculated the time interval between the initiation of the LFP response and the occurrence of the sensory-evoked spikes.

## Results

To examine the transformation of whisker interactions with surfaces into cortical neuronal discharges, we replayed receptive whisker sensing of different surfaces by covering the face of a rotating cylinder with several grades of sandpaper with varying degrees of coarseness (Lottem and Azouz, 2008). The cylinder face was placed orthogonally to the vibrissae such that they rested upon it (Figure 1A). The experimental goal was to collect records of the movement of whiskers across surfaces and use them as a stimulus set to probe the neuronal representation of surface coarseness (Lottem and Azouz, 2009). The current study examined several measures of cortical neuronal responses to different textures. We recorded local field potentials (LFPs) and spikes with multi-site silicon probe electrodes from vS1 neurons. Single-neuron spike trains were obtained through spike sorting (see Methods) to separate the spike trains originating from distinct neurons. The resulting sample consisted of 212 multi-unit recordings encompassing all layers, with the following distribution: 64 in the layers 2–3, 57 in layer 4, and 91 in the layers 5–6. In our study, we examined the responses to up to five textures. We quantified various neuronal parameters, including discharge rates, spike count correlations, neuronal synchronization, and neuronal responses to SSE in response to different surfaces.

An example of whisker motion for two textures is shown in Figure 1B. We quantitatively evaluated the changes in whisker angle and curvature caused by texture coarseness. We first calculated the whiskers’ position and the curvature SD of each measured whisker vibration in response to all studied textures to quantify these changes (the SD of these signals was calculated throughout stimulus duration: 500 ms). We quantified the range of whisker vibration in response to the different textures. As shown in Figure 1C for the recording in Figure 1B, a relationship was observed between texture coarseness and whisker response characteristics. Coarser and finer surfaces are expressed by higher and lower response SD values (Gugig et al., 2020). These results were consistent across recordings for all whiskers (Figure 1D; *n* = 17) and suggest that the amplitude of SSE changes globally due to surface coarseness (Wolfe et al., 2008; Gugig et al., 2020).

PSTHs for paired cortical neuronal responses to the different textures are shown in Figure 1E. These examples corroborate our previous findings that different cortical neurons respond differently to various surfaces (Garion et al., 2014). We quantified the neuronal responses in Figure 1F, demonstrating that the first neuron reduces its firing rate monotonically as a function of surface coarseness. In contrast, the second neuron shows selectivity to P400.

We tested whether a global characteristic of SSEs could provide a code for surface coarseness. Such an SSE code is plausible given that sharp, high-acceleration events effectively drive spikes in the somatosensory cortex (Shoykhet et al., 2000; Arabzadeh et al., 2005; Wolfe et al., 2008; Sharma and Azouz, 2022). The pattern of SSEs will thus likely be encoded in the cortex. We used acceleration to identify these events (Wolfe et al., 2008). We compared acceleration events on four sandpaper textures. This measurement was performed for the C2, C3, D2, D3 whiskers (*n* = 17). For this analysis, an *acceleration event* was defined as any acceleration peak that crossed a defined threshold. We normalized each signal to the Z-score, so our threshold was reported in units of SD. We defined low-acceleration events as events occurring at a threshold of 0.05–0.25 SD. In contrast, high-acceleration events occurred at a threshold above 0.5 SD (Wolfe et al., 2008). We calculated the ratio between the two and found that high-acceleration events may systematically occur more frequently on rougher surfaces (Figure 1H). These findings emphasize a graded association between texture coarseness and the ratio of low to high acceleration events. However, their explanatory power remains partial in accounting for the firing rates observed in the neurons during this session.

We characterized the SSE underlying the spikes to investigate further the transformation of whisker motion into cortical neuronal discharges. Figure 1G illustrates the distribution of SSE amplitudes for all the spikes in the neurons presented in Figures 1D,E. Notably, the number of events closely corresponds to the firing rates. We quantified this relationship by calculating the Pearson correlation coefficient (PCC), which yielded a value of 0.92 for the neurons in Figures 1E,G. Across all neurons, the average PCC was consistently 0.89.

Interestingly, the distribution of SSE amplitudes for the neuron in Figures 1E,G, upper panels exhibits a bimodal distribution in the upper panels, while the neuron shown in the lower panels displays an unimodal distribution in the lower panels. These distinct patterns of SSE amplitudes, when compared to firing rates in response to different textures (Figure 1G), suggest differential changes in the two separate neuronal response characteristics. Later, we will delve into a more comprehensive analysis of these phenomena and discuss their relevance to additional coding strategies in subsequent (Figures 2–5).

### Texture-dependent spike timing correlations

To understand whether cortical neuronal correlations encode information about surface coarseness, we conducted recordings from a total of 270 neuronal pairs. Figure 2A illustrates the paired cortical neuronal responses to various textures. These responses are visually represented using established peri-stimulus time histograms (PSTHs), showcasing how neurons react to four distinct textures. To delve into the temporal synchronization between these neuronal pairs, we computed the cross-correlation histograms (CCHs), as depicted in Figure 2B. These CCHs allow us to explore and quantify the degree of synchronization between the spike trains of these paired neurons.

We generated corresponding pseudorandom spike trains for each pair of neurons to assess the statistical significance of temporal synchronization. As seen in Figure 2B, the CCHs for simultaneously recorded neurons (as indicated in the upper and lower panels of Figure 2A) exhibited distinct peaks centered on zero time lag, consistent with synchronized pairs.

Notably, for this specific neuronal pair, the CCH exhibited variability dependent on surface coarseness, with the peak of the CCH differing among the various textures. We calculated the SR to measure this synchrony quantitatively. This involved dividing the magnitude of the original CCH by the confidence limit, which was determined using the criteria derived from pseudorandom spike trains (please refer to the Methods section). An SR value >1 was considered statistically significant.

We summarized the incidence of significant response synchronizations for all neurons taken from the same tetrode under all conditions in Figure 2D. Among the 212 pairs analyzed, 110 (52%) displayed significant temporal correlations in their spike trains, as indicated by SR values >1. Figure 2C illustrates the relationship between surface coarseness, discharge rates of the two recorded neurons, and their respective SR values. These examples highlight that cortical neurons exhibit changes in neuronal synchrony linked to surface coarseness. These findings underscore that neuronal synchronization is a prevalent and robust feature of stimulus-evoked activity (refer to Figures 6 7 for statistical analysis encompassing all neurons).

The SR values represent the ratio of synchronous spikes to what would occur by chance, meaning that each neuron’s responses consist of synchronous and asynchronous spikes. To distinguish between these, we defined all coincident spikes falling within the significant temporal window determined by the CCH width for each pair of neurons (Figure 2B) as synchronous. In contrast, all other spikes were categorized as asynchronous. An interesting pattern emerged when we examined the amplitudes of the SSE underlying all the spikes in these neurons. As depicted in Figure 2E, the distribution displayed a bimodal distribution, where asynchronous and synchronous spikes formed significantly separate peaks. This indicates that the SSE amplitudes associated with synchronized spikes were consistently larger than those linked to asynchronous spikes across all neurons (Sharma and Azouz, 2022).

We compared their distributions to investigate the impact of different SSE magnitudes on neuronal responses. In Figure 2E, we illustrate that, for the first neuron, the number of asynchronous SSE for P600 exceeded that of SSEs for P220, resulting in higher firing rates (green vs. blue; Figure 2C). Conversely, the number of synchronous SSEs was more pronounced for P220 than for P600 (red vs. pink). This pattern held for the second neuron as well. In this example, these neurons exhibited a preference for P600 in terms of firing rates, whereas they leaned towards P220 in terms of their degree of synchrony.

We computed several relationships to quantify the transformation of whisker vibrations into neuronal discharges across all neurons. First, we determined the Pearson correlation coefficient (calculated for each neuron, for each trial) between low-and high-acceleration event ratios (as shown in Figure 1H) and firing rates. This correlation was found to be relatively low (0.32 ± 0.18; depicted as the orange bar in Figure 2F). Second, we calculated the correlation coefficient between the number of asynchronous SSE and firing rates, revealing a relatively high correlation (0.86 ± 0.05, represented by the blue bar in Figure 2F). Third, we computed the correlation between the number of synchronized SSE and SR values, resulting in a correlation coefficient of 0.78 ± 0.18. These findings suggest that the proportion of high-magnitude SSE cannot fully explain neuronal firing rates. Moreover, the different aspects of neuronal responses vary differentially in response to surface coarseness.

### Texture-dependent local stick–slip event properties

Interactions between the whiskers and the environment lead to frictional movement and induce whisker bending, vibrations, and brief, discrete micromotions known as SSEs (Wolfe et al., 2008; Diamond et al.,2008a; Lottem and Azouz, 2009; O'Connor et al., 2010; Zuo et al., 2011; Chen et al., 2015; Campagner et al., 2018). Here, we examined whether surface coarseness impacts the transformation of SSEs into neuronal discharges. An example of whisker motion across P220 sandpaper is shown in Figure 3A, revealing multiple brief, high-acceleration events during whisker motion. Subsequently, we investigated the impact of synchronous and asynchronous spikes on tactile transformation. All coincident spikes occurring within the significant temporal window determined by the CCH width (see Methods) were designated synchronous and colored red. In contrast, all other spikes were designated asynchronous and colored black (Figure 3A, upper panels). The trace shows that the synchronized spikes are interspersed with spikes that do not exhibit temporal correlation.

Upon analyzing the neuronal response, we observed a correlation between high-velocity events and concurrent occurrences of significant negative deflections in both the LFP and the neuronal discharge. As we have shown previously (Sharma and Azouz, 2022), SSEs drove each spike, and different aspects of these neuronal discharges convey information regarding the magnitude of an SSE. We measured the Spike-Triggered Average (STA) of both synchronous and asynchronous spikes to determine if there were differences in their local transformations. In Figure 3B, you can see that the STAs obtained from asynchronous spikes (represented by the blue and red lines) were smaller and had higher variability. On the other hand, the STAs calculated from synchronous spikes (illustrated by the purple lines) were larger and showed less variability.

To examine whether surface coarseness affects the transformation of SSEs into spikes, we investigated the relationship between SSE amplitudes in the different textures and various aspects of neuronal discharges. An example of this analysis for paired neuronal recording is shown in Figures 3C,D. To begin with, we investigated the correlation between SSE amplitudes and discharge probability in the two types of spikes. In this analysis, any peak that surpassed a predetermined threshold was considered an SSE without distinguishing between stick and slip events (refer to the Methods section for more details). The examination focused on the initial 20 msec following each SSE. Results showed that the discharge probability of asynchronous spikes gradually increased with higher thresholds, suggesting their ability to reliably convey the magnitude of SSEs (illustrated by examples such as P600 and P220 in Figure 4C). In contrast, synchronous spikes displayed an almost all-or-none response pattern once they surpassed the higher threshold.

We computed the mean and SD values of the slopes and shifts in these response curves to quantify the distinctions between asynchronous and synchronous spikes for various textures in all neurons. Our findings revealed that asynchronous spikes altered their discharge probability with SSE amplitude, as evidenced by the smaller slope values. Conversely, synchronous spikes exhibited a steep rise in discharge probability with increasing SSE amplitude. Furthermore, with increasing surface coarseness, there was an observed increase in the slope for asynchronous spikes and a rightward shift in the curves for both asynchronous and synchronous spikes (Figure 3E). This effect was observed in 0.85 of significant neurons for asynchronous spikes and in 0.97 for synchronized spikes, highlighting the correlation between surface coarseness and these response characteristics.

Second, we investigated the relationship between SSE amplitudes and the neuronal discharge rates in the two groups of spikes, calculating firing rates over 30 ms. We detected a linear relationship between the neuronal discharge rates and the SSE amplitude for the asynchronous spikes (Figure 3D). Comparing all neurons revealed that the asynchronous spikes conveyed the SSE amplitude through their firing rates. In contrast, the synchronous spikes did so poorly, with 98 and 5% of asynchronous and synchronous neurons exhibiting this phenomenon. Increasing surface coarseness resulted in an increase in the slope for asynchronous spikes and a rightward shift in the asynchronous spike curve (Figure 3E). Together, these findings suggest that surface coarseness plays a role in influencing the transformation of SSE amplitudes into discharge probabilities and firing rates.

In a previous study (Sharma and Azouz, 2022), we showed that the response latency for synchronous spikes is defined as the relative interval between network activity and spikes, and the relative spike timing between synchronous spikes depends on SSE amplitude. Here, we examined whether these response characteristics are influenced by surface coarseness. First, we calculated the relationship between SSE amplitude and spike latency. We examined synchronous spikes that show this dependency and compared this dependence across different textures. For each spike, we detected its underlying SSEs and quantified the timing of each SSE by defining the peak value that crossed the threshold at the mean ± 3 SD (Figure 4A). The examples shown in Figure 4 are for the same neurons as in Figure 3. Figure 4B shows the dependence of synchronized spike latency on SSE amplitude for two textures. Each point represents an average of multiple points. These figures show that the spike latency in synchronous spikes was linearly dependent on the SSE amplitude. We then quantified this relationship by calculating the normalized slopes for all neurons with synchronous spikes for these textures, normalizing SSE slopes to the z score. We found that the synchronous spikes showed a significant latency dependence on the SSE amplitude in 89% of the neurons (Figure 4G; *t* (126) = 9.3, *p* = 0.000127).

Second, we examined the influence of SSE amplitude on the time interval between the sensory-evoked commencement of the LFP and associated spikes. We calculated its second derivative to define the sensory-evoked LFP starting point (Figure 4C; see Methods). We examined synchronous spikes that exhibited this dependency and compared such dependence across different textures. Figure 4D shows the dependence of the LFP-spike latency on SSE amplitude for two surfaces in synchronous spikes. These results demonstrate that the LFP-spike latency for synchronous spikes depended on the SSE amplitude. We found that the synchronous spikes exhibited a significant latency dependence on the SSE position amplitude in 80% of the neurons (Figure 4H; *t* (126) = 15.3, *p* = 0.000012).

In our final analysis, we explored the impact of SSE amplitude on the level of spike synchrony, as reflected by the inter-neuronal inter-spike interval (ISI) (refer to Figure 4E and the Methods section for details). Figure 4F provides an illustrative example of this relationship, revealing that larger SSE amplitudes were associated with smaller ISIs. Moreover, this dependence could be accurately described by an exponential decay function (as depicted in Figure 4F). These data show the dependence of ISI on SSE amplitude and highlight a decrease in this slope for coarser surfaces (Figure 4I; *t* (126) = 19.1, *p* = 2.3e^−4^), with 90.2% of neurons exhibiting this significant dependence. This indicates that the distinct attributes of asynchronous and synchronous spike responses are influenced in varying ways by surface coarseness. It suggests that textural variations uniquely impact different aspects of neuronal firing related to asynchronous and synchronous spikes.

### Comparisons of the different coding strategies

Figure 5 shows an example of the different coding strategies as a function of surface coarseness. Here, we recorded from a pair of neurons shown in Figures 3 4, presenting the firing rate of each neuron (Figure 5A; red and blue traces) and their synchronous activity (SR values; purple trace) as a function of surface coarseness. These results indicated that the first neuron exhibited a preference for the P600 texture, whereas the second neuron exhibited a P220 preference. The synchronous activity of the two neurons also presented with a P600 preference. Figure 5B shows the dependence of the local asynchronous number of spikes and probability slope on surface coarseness for the same neurons. These two response characteristics exhibited a preference for P600. Finally, Figure 5C highlights the dependence of local synchronous response characteristics on surface coarseness, specifically a preference for the P220 texture. Our results thus suggest that changes in texture coarseness lead to complex neuronal responses (Figures 1–5), which implies that cortical neurons may be selective for specific textures through texture selectivity (Garion et al., 2014). Notably, distinct coding strategies may present different texture selectivity.

We next examined several surface coarseness-dependent responses to compare three different coding strategies: selectivity, clustering, and similarity. Cortical neuron texture selectivity was assessed using a texture selectivity index (SI) (Figures 4A,B; see Methods). First, we calculated the average firing rate across 75 trials for four different textures and computed the SI to quantify the statistical significance of texture selectivity. Second, we shuffled the firing rate of 75 trials between four surfaces 500 times and calculated the SI for each shuffling. Then, we calculated the mean + 3SD for the 500 points of the SI data distribution (Figure 6E). If the calculated SI of the original data is higher than the shuffled SI (mean + 3SD), the SI was considered statistically significant. We previously reported that more than 80% of the recorded cortical neurons are texture-selective (Garion et al., 2014).

When we plotted the firing rate and SR associated with the different textures of paired neurons (Figures 1–6), we did not observe a linear relationship between the firing rate and SR with texture coarseness. This suggests texture selectivity for firing rate, temporal synchronization, and local characteristics (the mean value across all local features). To quantify these complex relationships, we divided the neural responses as a function of surface coarseness into four categories (Figure 6C lower panels; see Methods section): (1) Up - neurons presenting a significant monotonic increase. (2) Down - neurons presenting a significant monotonic decrease. (3) Tuned - neurons exhibiting a preference for a specific texture (reduction and increase). (4) No change - neurons that did not show any significant changes. This analysis revealed that most neurons show texture selectivity (Figure 6C, middle panel) (0.82, 0.68, and 0.89 for firing rate, temporal synchronization, and local features, respectively). These results suggest that ~80% of the cortical neurons preferred a specific texture in global and local features. We divided these neurons according to their preferred surface. We found neurons that preferentially responded to each examined texture (Figure 6D). These findings suggest that cortical neurons can represent a broad spectrum of surface coarseness. This implies that these neurons are adept at encoding and responding to various textural coarseness within their signaling.

To further examine texture selectivity, we calculated the texture SI for all coding strategies (Figure 6E). We measured respective SI values of 0.35 ± 0.19, 0.56 ± 0.18, and 0.52 ± 0.12 (mean ± SD) for the firing rate, synchronization, and local features, respectively. Thus, synchronous activity and local features show higher texture selectivity than firing rates (*F* (2,126) = 25.4, *p* = 0.00235). Finally, to examine the capacity of the different coding strategies to discriminate between the various surfaces, we calculated the ROC-based AUC values for all textures. The result indicated a significantly higher mean AUC value for local features (0.78) as compared to synchronization (0.61) and firing rate (0.57) (Figure 6F) (*F* (2,126) = 18.3, *p* = 0.00122). Together, these findings imply that the various coding strategies exhibit differences in their selectivity for textures and their capability to discriminate between different textures. This suggests a variability in how effectively each strategy can distinguish and respond to diverse textures.

We additionally assessed the similarity between the different coding strategies to determine whether different coding strategies exhibit the same surface coarseness preference. We divided the neurons into three categories: (1) Similar coding strategies show similar dependence on surface coarseness. Regarding firing rates and synchrony, the preferred texture firing rates for two neurons correspond to the preferred texture for synchronous activity (Figure 7A); (2) partially different - different coding strategies show partially similar dependence on surface coarseness. Only one neuron’s preferred texture firing rates correspond to the preferred texture for synchronous activity (Figure 7B); (3) different coding strategies show dissimilar dependence on surface coarseness. None of the neurons’ preferred texture firing rates correspond to the preferred texture for synchronous activity (Figure 7C).

Initially, we determined the preferred texture for each coding strategy (Figure 6). Based on the similarity in surface coarseness preferences, we then divided the analyzed neurons into these three categories. For each group, we calculated Pearson correlation coefficient values between the plots, revealing that the proportions of cortical neurons in each of these categories for the relationship between firing rates and synchrony were: Similar - 0.2 (0.5), partially different - 0.35 (0.56), and different - 0.55 (−0.45) (Figure 7D), where the values in parentheses are the average Pearson correlation coefficients among the different plots. We found the following distribution for the relationship between firing rates and local features: Similar - 0.1 (0.35), partially different - 0.38 (0.58), and different - 0.52 (−0.01). We repeated this analysis for the relationship between synchrony and local features, yielding the following group proportions: Similar - 0, partially different - 0.25 (0.66), and different - 0.75 (0.1). These findings imply that the three coding strategies represent surface coarseness independently within the same neurons. This suggests distinct and separate ways in which surface coarseness is encoded by these strategies within the neural network.

Finally, a previous imaging study has shown that the cortical neurons cluster spatially following their texture selectivity (Garion et al., 2014). To examine the degree of spatial clustering of texture selectivity of neurons recorded from the same site (<150 μm), we devised a similarity measure between adjacent neurons, termed the *Similarity value* (see Methods). We calculated this Similarity value as the number of neurons selective to the same texture divided by the number of neurons in a particular cluster. A similarity value closer to 1 indicates that all neurons in the group have the same preferred surface. We found that for firing rates, the Similarity value was 0.72 ± 0.21, which was well above the significance level of 0.49 (see Methods for details regarding the calculation of the significance level), indicating a high degree of texture similarity between adjacent neurons (Figure 7E, blue bar). In several cases (*n* = 25), we calculated the Similarity value for synchronous neurons when we recorded from synchronous triplet and quadruplet neurons. We found that this Similarity value was 0.52 ± 0.15, indicating a low degree of texture similarity between adjacent neurons (Figure 7E, purple bar). We repeated the same analysis with local coding and measured a mean Similarity value of 0.58 ± 0.07 across all local features, suggesting that local features of neurons do not show spatial clustering. These findings suggest a distinction in the spatial arrangement associated with these three coding strategies, highlighting unique organizational patterns among them.

## Discussion

In the current study, we explored the transformation of tactile inputs into cortical neuronal discharges by monitoring the kinematic and kinetic characteristics associated with whisker motion across textured surfaces and concurrently recording from a small population of cortical neurons. SSEs are a prominent feature of whisker-surface interactions. Recent studies have shown that the kinematic profiles of SSEs carry textural information (Ritt et al., 2008; Wolfe et al., 2008) and are encoded by neurons on the ascending tactile pathway (Simons, 1978; Pinto et al., 2000; Jones et al., 2004; Arabzadeh et al., 2005; Kerr et al., 2007; Stuttgen and Schwarz, 2008, 2010; Jadhav et al., 2009; Jadhav and Feldman, 2010; Crochet et al., 2011; Waiblinger et al., 2013, 2015; Allitt et al., 2017; Isett et al., 2018). The magnitude and frequency of these events were correlated with texture, with rougher sandpapers eliciting a larger amplitude of SSE position and forces, as well as an increase in their number (Figure 1H; Wolfe et al., 2008), as compared to smoother surfaces (Figures 1C,D; Wolfe et al., 2008; Gugig et al., 2020). These changes may result from the relationship between high-and low-acceleration events (Figure 1H; Wolfe et al., 2008). The association between SSE magnitude, forces, and texture may result from greater friction between whiskers and rougher surfaces. These changes reflected the global interaction between surfaces and whiskers. Changes in surface coarseness also resulted in a modification in local interactions between the whiskers and surfaces (Poulet and Petersen, 2008; Oladazimi et al., 2018; Gugig et al., 2020; Sharma and Azouz, 2022). Since SSEs *constitute a significant* factor contributing to most spike generation in vS1 neurons (Arabzadeh et al., 2005; Lottem and Azouz, 2008; Wolfe et al., 2008; Lottem and Azouz, 2009) during whiskers-surface interactions (Figures 3, 4), it was hypothesized that the variations in the characteristics of SSEs as a function of surface coarseness are directly related to the mean neuronal firing rates (Figure 1). However, these changes in whisker surface interactions, shown here and elsewhere to be gradual and surface coarseness-dependent, could not explain cortical selective neuronal responses (Figure 1). We found that surface coarseness is encoded in the mean firing rate of vS1 neurons (Arabzadeh et al., 2003; von Heimendahl et al., 2007; Wolfe et al., 2008; Jadhav et al., 2009). These relationships indicate that most vS1 neurons exhibit a surface coarseness preference (Figures 1 5; Garion et al., 2014).

We have demonstrated that the magnitude and number of SSEs are components of the kinetic signature associated with various textures. However, coding these parameters by vS1 neurons is not a result of the direct transformation of whisker-surface interactions (Figure 2F, orange bar). Instead, texture-specific changes in firing rate depend on neural sensitivity to SSE kinetic and kinematic characteristics (Garion et al., 2014; Sharma and Azouz, 2022). Moreover, the direct relationship between SSEs and spike discharges argues against the notion that cortical neuronal discharges result from the temporal integration of the vibrotactile signal within relatively extended ranges. Thus, our results do not support a model in which surface coarseness is transformed into the mean firing rate of vS1 neurons (Arabzadeh et al., 2003; von Heimendahl et al., 2007; Wolfe et al., 2008; Jadhav et al., 2009), indicating that it is related to the mean speed or total power of surface-induced whisker vibrations (Arabzadeh et al., 2005, 2006; von Heimendahl et al., 2007).

Another novel and plausible coding strategy outlined in the current study relies on precise spike timing through the spatiotemporal synchronization of neuronal assemblies. We found that neuronal synchronization is a prevalent and robust feature of spontaneous and stimulus-evoked activity (Zhang and Alloway, 2004; Zhang and Alloway, 2006). We found that the tactile stimulus-driven neuronal discharge of nearby neurons consists of a mixture of synchronous and asynchronous spikes. This temporal synchronization is stimulus-driven (Sharma and Azouz, 2022). We further uncovered novel evidence that the degree of synchronization changes as a function of surface coarseness (Figures 2A–C). These changes show surface coarseness preference that manifests in these firing rates. Although this measure of neuronal synchrony indicates local temporal interactions, the SR value we used here reflects the ratio between the number of events resulting in synchronous spikes and that expected by chance. Thus, one may argue that this measure reflects global coding (Figures 2E,F, blue and red bars) and may be attributable to variations in the number and magnitude of SSEs.

By measuring concurrent neuronal activity and whisker movement in response to multiple surfaces, we found that variations in surface coarseness resulted in changes in SSE characteristics. These changes, designated here as local changes, reflect the complex local transformation of the kinetic and kinematic characteristics of SSEs to yield neuronal discharges (Figures 4 5). These surface coarseness-dependent changes were expressed in the distribution of SSEs underlying spike discharges. Changes in surface coarseness resulted in a shift in the amplitude of spike-generating SSEs (Figures 1G,I, 2E). By assessing the level of synchrony between neighboring neurons, we observed that the neuronal responses to textures exhibited a combination of synchronous and asynchronous spikes, which were intermingled (Figure 2E; Sharma and Azouz, 2022). Once we separated the synchronous and asynchronous spikes, we differentiated their underlying SSEs and successfully identified coexisting tactile information streams and corresponding coding strategies (Lankarany et al., 2019). Within a short time frame, asynchronous and synchronous spikes convey an unexpected level of detail regarding SSE magnitude via multiple channels, including spike rates and probability in asynchronous spikes (Figures 3^4^). Furthermore, synchronous spikes convey SSE magnitude through the precise timing of spikes between and within neurons (Figure 4). Our data thus indicate that the relationship between SSE magnitude and the different features of neuronal responses can serve to discriminate between different textures (Figures 4 5).

We have shown that most neurons exhibit a surface coarseness preference across all coding strategies (Figure 6C) and that these neurons show a preference for all textures (Figure 6D). By using AUC values and the selectivity index, we further determined that the tactile evidence carried by local stick–slip event properties was superior to other coding strategies concerning discrimination among different textures (Figures 6E,F). Moreover, comparisons of the specific preferred surfaces in the same neurons through these various coding strategies revealed that most neurons present with differing degrees of texture selectivity through these different coding strategies (Figures 7A–D). Our findings indicate that the discrepancy between these coding strategies in transmitting preferred texture information thus stems from their sensitivity to different tactile features.

Whisker-surface interactions are likely to be modulated in response to environmental conditions, tasks, and the motivation of the animal (Carvell and Simons, 1995; Berg and Kleinfeld, 2003; Sachdev et al., 2003; Towal and Hartmann, 2006; Mitchinson et al., 2007; Hill et al., 2008; Towal and Hartmann, 2008; Grant et al., 2009; Zuo et al., 2011; Voigts et al., 2015), as well as considerable variations in stimulus configuration, whisker velocity, head movements, and object distances (Carvell and Simons, 1990; von Heimendahl et al., 2007; Diamond et al.,2008b; Wolfe et al., 2008; Diamond, 2010; Morita et al., 2011; Voigts et al., 2015). These changes can lead to considerable changes in sensory signals (Lichtenstein et al., 1990; Shoykhet et al., 2000; Szwed et al., 2003) and may differentially influence the various coding strategies, changing associated surface coarseness preferences.

These results suggest that cortical neurons may have access to a more detailed, dynamic description of tactile inputs than initially assumed. These coding strategies may enable spike trains to convey stimulus information through multiple complementary channels, each corresponding to a different aspect of the tactile world and its variations, thereby better coping with a dynamic and complex tactile environment.

### Methodological considerations

To explore the transformation of the tactile features of whisker vibrations into cortical neuronal activity, we used receptive sensing in which the whiskers are stationary and the surfaces move. Rats actively sweep their whiskers across surfaces to locate and distinguish objects in the animals’ immediate sensory environment (Welker, 1964; Carvell and Simons, 1990; Sachdev et al., 2001; Bermejo et al., 2002; Berg and Kleinfeld, 2003; Knutsen et al., 2005). In addition, active whisking is often associated with head and body movements (Carvell and Simons, 1995; Brecht et al., 1997; Mitchinson et al., 2007; Ritt et al., 2008; Towal and Hartmann, 2008). Moreover, rodents often forego whisking, relying solely on passive movement of their whiskers instigated by body and head movements. Specifically, they use their vibrissae but do not whisk as they maintain contact with walls and surfaces while running.

The behavioral paradigms used to study texture discrimination extensively influence how rats use their whiskers to sense the tactile environment. In head-fixed animals, the only way to sense the surfaces is to whisk against them. However, to our knowledge, a quantitative examination has yet to be published regarding the influence of whisking strategies on texture discrimination under these conditions. That may be due to stable conditions where the surfaces are located at a constant location and distance, influencing whisking strategies and making perception and discrimination less complex (Wolfe et al., 2008; Jadhav and Feldman, 2010).

In free-behaving animals, it has been shown that they develop a purposive whisking strategy for whisker-surface interactions that is information-seeking to perceive and discriminate between different textures. Thus, whisking behavior is related mainly to gathering tactile information, whereas discrimination performance appears to be more closely associated with the details of whisker–surface interactions (Zuo et al., 2011; Zuo and Diamond, 2019). Finally, it has been shown recently that free-behaving rats can discriminate fine tactile patterns while running without whisking (Kerekes et al., 2017).

The present study primarily examined the transformation of whisker-surface interactions into cortical neuronal activity. While we use anesthetized rats in receptive sensing mode, our results are similar to those in awake-behaving animals in many respects. The occurrence and magnitude of SSEs are encoded by time-locked spikes in vS1 ensembles, with texture-related sequences of SSEs encoded by multiple coding strategies constrained by the intrinsic dynamics of whisker circuits and synapses. The occurrence of discrete SSEs related to texture, observed here, suggests coexisting coding for texture during active sensation while awake.

### Texture selectivity in cortical neurons

A hallmark of the sensorimotor system is that tactile features are organized according to maps whereby the functional role of a neuron and its tuning to stimulus properties can be predicted by its location. Several properties of tactile stimuli have been spatially identified in the somatosensory whisker system. First, whisker somatotopy is perhaps the most prominent property of the whisker pathway organization (Woolsey and Van der Loos, 1970; Welker and Woolsey, 1974). Additionally, a map of directional selectivity has been observed in anesthetized and awake rodents performing active sensing (Andermann and Moore, 2006; Kerr et al., 2007; Peron et al., 2015). Another spatial feature of whisker stimuli is the degree of correlated motion across multiple whiskers (Estebanez et al., 2016). In contrast, the distribution of response selectivity for whisker angle, curvature, kinematic features, and distance to a wall found clear evidence favoring a salt-and-pepper distribution of feature selectivity (Peron et al., 2015; Sofroniew et al., 2015; Martini et al., 2017). Studies of selectivity to texture coarseness in rats, tested under electrical whisking conditions, found that neurons preferring the same texture tend to cluster together within the Barrel (Garion et al., 2014). In the current study, we use receptive sensing to support our previous finding that cortical neurons exhibit surface coarseness preference through different coding strategies (Figure 6).

Cortical neurons display spatial clustering according to their preferred texture selectivity. However, this clustering is restricted to firing rates only. Examination of the spatial organization through the two other coding strategies revealed no spatial clustering (Figure 7E). Thus, our findings suggest that surface coarseness preference is an inherent feature of tactile transformation. These transformations may reflect a unique combination of kinetic and kinematic features for each texture. These combinations manifest themselves through the different coding strategies in which firing rates primarily represent global parameters such as the number and magnitude of SSEs. Synchrony coding reflects the number and magnitude of SSEs that resulted in synchronized spikes. These results suggest a differential role for these different coding strategies in which each neuron participates in overlapping populations coding different attributes of whisker-mediated sensory signals (Derdikman et al., 2003; Komiyama et al., 2010; Rothschild et al., 2010; Ko et al., 2011). A comprehensive understanding of how neuronal networks are organized will thus need to consider how different neurons convey sensory and other relevant signals under different tactile and behavioral conditions.

## Data availability statement

The raw data supporting the conclusions of this article will be made available by the authors, without undue reservation.

## Ethics statement

The animal study was approved by Ben-Gurion University of the Negev. The study was conducted in accordance with the local legislation and institutional requirements.

## Author contributions

HS: Data curation, Formal analysis, Methodology, Software, Writing – review & editing. RA: Conceptualization, Funding acquisition, Project administration, Resources, Supervision, Writing – original draft, Writing – review & editing.

## Footnotes

^1^http://redishlab.neuroscience.umn.edu/MClust/MClust.html

## References

[ref1] Allitt B. J. Alwis D. S. Rajan R. (2017). Laminar-specific encoding of texture elements in rat barrel cortex. J. Physiol. 595, 7223–7247. doi: 10.1113/JP274865, PMID: 28929510 PMC5709323

[ref2] Andermann M. L. Moore C. I. (2006). A somatotopic map of vibrissa motion direction within a barrel column. Nat. Neurosci. 9, 543–551. doi: 10.1038/nn1671 16547511

[ref3] Arabzadeh E. Panzeri S. Diamond M. E. (2006). Deciphering the spike train of a sensory neuron: counts and temporal patterns in the rat whisker pathway. J. Neurosci. 26, 9216–9226. doi: 10.1523/JNEUROSCI.1491-06.2006 16957078 PMC6674492

[ref4] Arabzadeh E. Petersen R. S. Diamond M. E. (2003). Encoding of whisker vibration by rat barrel cortex neurons: implications for texture discrimination. J. Neurosci. 23, 9146–9154. doi: 10.1523/JNEUROSCI.23-27-09146.2003, PMID: 14534248 PMC6740840

[ref5] Arabzadeh E. Zorzin E. Diamond M. E. (2005). Neuronal encoding of texture in the whisker sensory pathway. PLoS Biol. 3:e17. doi: 10.1371/journal.pbio.0030017, PMID: 15660157 PMC544542

[ref6] Azouz R. Gray C. M. (2000). Dynamic spike threshold reveals a mechanism for synaptic coincidence detection in cortical neurons in vivo. Proc. Natl. Acad. Sci. U. S. A. 97, 8110–8115. doi: 10.1073/pnas.130200797, PMID: 10859358 PMC16678

[ref7] Azouz R. Gray C. M. (2003). Adaptive coincidence detection and dynamic gain control in visual cortical neurons in vivo. Neuron 37, 513–523. doi: 10.1016/S0896-6273(02)01186-8, PMID: 12575957

[ref8] Berg R. W. Kleinfeld D. (2003). Rhythmic whisking by rat: retraction as well as protraction of the vibrissae is under active muscular control. J. Neurophysiol. 89, 104–117. doi: 10.1152/jn.00600.2002, PMID: 12522163

[ref9] Bermejo R. Vyas A. Zeigler H. P. (2002). Topography of rodent whisking-I. Two-dimensional monitoring of whisker movements. Somatosens. Mot. Res. 19, 341–346. doi: 10.1080/0899022021000037809, PMID: 12590835

[ref10] Birdwell J. A. Solomon J. H. Thajchayapong M. Taylor M. A. Cheely M. Towal R. B.. (2007). Biomechanical models for radial distance determination by the rat vibrissal system. J. Neurophysiol. 98, 2439–2455. doi: 10.1152/jn.00707.2006, PMID: 17553946

[ref11] Brecht M. Preilowski B. Merzenich M. M. (1997). Functional architecture of the mystacial vibrissae. Behav. Brain Res. 84, 81–97. doi: 10.1016/S0166-4328(97)83328-1, PMID: 9079775

[ref12] Brette R. (2012). Computing with neural synchrony. PLoS Comput. Biol. 8:e1002561. doi: 10.1371/journal.pcbi.1002561, PMID: 22719243 PMC3375225

[ref13] Campagner D. Evans M. H. Loft M. S. E. Petersen R. S. (2018). What the whiskers tell the brain. Neuroscience 368, 95–108. doi: 10.1016/j.neuroscience.2017.08.005 28843998

[ref14] Carvell G. E. Simons D. J. (1990). Biometric analyses of vibrissal tactile discrimination in the rat. J. Neurosci. 10, 2638–2648. doi: 10.1523/JNEUROSCI.10-08-02638.1990, PMID: 2388081 PMC6570272

[ref15] Carvell G. E. Simons D. J. (1995). Task-and subject-related differences in sensorimotor behavior during active touch. Somatosens. Mot. Res. 12, 1–9. doi: 10.3109/08990229509063138, PMID: 7571939

[ref16] Chen J. L. Margolis D. J. Stankov A. Sumanovski L. T. Schneider B. L. Helmchen F. (2015). Pathway-specific reorganization of projection neurons in somatosensory cortex during learning. Nat. Neurosci. 18, 1101–1108. doi: 10.1038/nn.4046, PMID: 26098757

[ref17] Clack N. G. O'Connor D. H. Huber D. Petreanu L. Hires A. Peron S.. (2012). Automated tracking of whiskers in videos of head fixed rodents. PLoS Comput. Biol. 8:e1002591. doi: 10.1371/journal.pcbi.1002591, PMID: 22792058 PMC3390361

[ref18] Cohen M. R. Kohn A. (2011). Measuring and interpreting neuronal correlations. Nat. Neurosci. 14, 811–819. doi: 10.1038/nn.2842, PMID: 21709677 PMC3586814

[ref19] Crochet S. Poulet J. F. A. Kremer Y. Petersen C. C. H. (2011). Synaptic mechanisms underlying sparse coding of active touch. Neuron 69, 1160–1175. doi: 10.1016/j.neuron.2011.02.022, PMID: 21435560

[ref20] Dan Y. Alonso J. M. Usrey W. M. Reid R. C. (1998). Coding of visual information by precisely correlated spikes in the lateral geniculate nucleus. Nat. Neurosci. 1, 501–507. doi: 10.1038/2217, PMID: 10196548

[ref21] Derdikman D. Hildesheim R. Ahissar E. Arieli A. Grinvald A. (2003). Imaging spatiotemporal dynamics of surround inhibition in the barrels somatosensory cortex. J. Neurosci. 23, 3100–3105. doi: 10.1523/JNEUROSCI.23-08-03100.2003 12716915 PMC6742298

[ref22] Diamond M. E. (2010). Texture sensation through the fingertips and the whiskers. Curr. Opin. Neurobiol. 20, 319–327. doi: 10.1016/j.conb.2010.03.004, PMID: 20403683

[ref23] Diamond M. E. von Heimendahl M. Arabzadeh E. (2008a). Whisker-mediated texture discrimination. PLoS Biol. 6:e220. doi: 10.1371/journal.pbio.0060220, PMID: 18752356 PMC2525693

[ref24] Diamond M. E. von Heimendahl M. Knutsen P. M. Kleinfeld D. Ahissar E. (2008b). 'Where' and 'what' in the whisker sensorimotor system. Nat. Rev. Neurosci. 9, 601–612. doi: 10.1038/nrn2411 18641667

[ref25] Estebanez L. Bertherat J. Shulz D. E. Bourdieu L. Léger J.-F. (2016). A radial map of multi-whisker correlation selectivity in the rat barrel cortex. Nat. Commun. 7:13528. doi: 10.1038/ncomms13528, PMID: 27869114 PMC5121329

[ref26] Ferezou I. Haiss F. Gentet L. J. Aronoff R. Weber B. Petersen C. C. H. (2007). Spatiotemporal dynamics of cortical sensorimotor integration in behaving mice. Neuron 56, 907–923. doi: 10.1016/j.neuron.2007.10.007, PMID: 18054865

[ref27] Friedberg M. H. Lee S. M. Ebner F. F. (1999). Modulation of receptive field properties of thalamic somatosensory neurons by the depth of anesthesia. J. Neurophysiol. 81, 2243–2252. doi: 10.1152/jn.1999.81.5.2243, PMID: 10322063

[ref28] Garion L. Dubin U. Rubin Y. Khateb M. Schiller Y. Azouz R.. (2014). Texture coarseness responsive neurons and their mapping in layer 2-3 of the rat barrel cortex in vivo. elife 3:e03405. doi: 10.7554/eLife.03405, PMID: 25233151 PMC4166033

[ref29] Grant R. A. Mitchinson B. Fox C. W. Prescott T. J. (2009). Active touch sensing in the rat: anticipatory and regulatory control of whisker movements during surface exploration. J. Neurophysiol. 101, 862–874. doi: 10.1152/jn.90783.2008, PMID: 19036871 PMC2657060

[ref30] Gray C. M. (1999). The temporal correlation hypothesis of visual feature integration: still alive and well. Neuron 24, 31–47. doi: 10.1016/S0896-6273(00)80820-X 10677025

[ref31] Gray C. M. Singer W. (1989). Stimulus-specific neuronal oscillations in orientation columns of cat visual cortex. Proc. Natl. Acad. Sci. 86, 1698–1702. doi: 10.1073/pnas.86.5.1698, PMID: 2922407 PMC286768

[ref32] Green D. M. Swets J. A. (1974). Signal detection theory and psychophysics. Huntington. New York: RE Krieger Pub. Co.

[ref33] Gugig E. Sharma H. Azouz R. (2020). Gradient of tactile properties in the rat whisker pad. PLoS Biol. 18:e3000699. doi: 10.1371/journal.pbio.3000699 33090990 PMC7608947

[ref34] Harris A. Z. Gordon J. A. (2015). Long-range neural synchrony in behavior. Annu. Rev. Neurosci. 38, 171–194. doi: 10.1146/annurev-neuro-071714-034111, PMID: 25897876 PMC4497851

[ref35] Hartmann M. J. Johnson N. J. Towal R. B. Assad C. (2003). Mechanical characteristics of rat vibrissae: resonant frequencies and damping in isolated whiskers and in the awake behaving animal. J. Neurosci. 23, 6510–6519. doi: 10.1523/JNEUROSCI.23-16-06510.2003, PMID: 12878692 PMC6740620

[ref36] Harvey M. A. Bermejo R. Zeigler H. P. (2001). Discriminative whisking in the head-fixed rat: optoelectronic monitoring during tactile detection and discrimination tasks. Somatosens. Mot. Res. 18, 211–222. doi: 10.1080/01421590120072204, PMID: 11562084

[ref37] Hebb D. O. Martinez J. L. Glickman S. E. (1994). The organization of behavior – a neuropsychological theory. Contemp. Psychol. 39, 1018–1020. doi: 10.1037/034206

[ref38] Hill D. N. Bermejo R. Zeigler H. P. Kleinfeld D. (2008). Biomechanics of the vibrissa motor plant in rat: rhythmic whisking consists of triphasic neuromuscular activity. J. Neurosci. 28, 3438–3455. doi: 10.1523/JNEUROSCI.5008-07.2008, PMID: 18367610 PMC6670594

[ref39] Hipp J. Arabzadeh E. Zorzin E. Conradt J. Kayser C. Diamond M. E.. (2006). Texture signals in whisker vibrations. J. Neurophysiol. 95, 1792–1799. doi: 10.1152/jn.01104.2005, PMID: 16338992

[ref40] Hires S. A. Pammer L. Svoboda K. Golomb D. (2013). Tapered whiskers are required for active tactile sensation. elife 2:e01350. doi: 10.7554/eLife.01350, PMID: 24252879 PMC3828597

[ref41] Isbister J. B. Reyes-Puerta V. Sun J. J. Horenko I. Luhmann H. J. (2021). Clustering and control for adaptation uncovers time-warped spike time patterns in cortical networks in vivo. Sci. Rep. 11:15066. doi: 10.1038/s41598-021-94002-0, PMID: 34326363 PMC8322153

[ref42] Isett B. R. Feasel S. H. Lane M. A. Feldman D. E. (2018). Slip-based coding of local shape and texture in mouse S1. Neuron 97, 418–433.e5. doi: 10.1016/j.neuron.2017.12.021, PMID: 29307709 PMC5773356

[ref43] Jadhav S. P. Feldman D. E. (2010). Texture coding in the whisker system. Curr. Opin. Neurobiol. 20, 313–318. doi: 10.1016/j.conb.2010.02.014, PMID: 20299205

[ref44] Jadhav S. P. Wolfe J. Feldman D. E. (2009). Sparse temporal coding of elementary tactile features during active whisker sensation. Nat. Neurosci. 12, 792–800. doi: 10.1038/nn.2328, PMID: 19430473

[ref45] Jones L. M. Lee S. Trageser J. C. Simons D. J. Keller A. (2004). Precise temporal responses in whisker trigeminal neurons. J. Neurophysiol. 92, 665–668. doi: 10.1152/jn.00031.2004 14999053 PMC2800049

[ref46] Kerekes P. Daret A. Shulz D. E. Ego-Stengel V. (2017). Bilateral discrimination of tactile patterns without whisking in freely running rats. J. Neurosci. 37, 7567–7579. doi: 10.1523/JNEUROSCI.0528-17.2017, PMID: 28663200 PMC6596651

[ref47] Kerr J. N. de Kock C. P. Greenberg D. S. Bruno R. M. Sakmann B. Helmchen F. (2007). Spatial organization of neuronal population responses in layer 2/3 of rat barrel cortex. J. Neurosci. 27, 13316–13328. doi: 10.1523/JNEUROSCI.2210-07.2007, PMID: 18045926 PMC6673403

[ref48] Kleinfeld D. Ahissar E. Diamond M. E. (2006). Active sensation: insights from the rodent vibrissa sensorimotor system. Curr. Opin. Neurobiol. 16, 435–444. doi: 10.1016/j.conb.2006.06.009, PMID: 16837190

[ref49] Kleinfeld D. Deschenes M. (2011). Neuronal basis for object location in the vibrissa scanning sensorimotor system. Neuron 72, 455–468. doi: 10.1016/j.neuron.2011.10.009, PMID: 22078505 PMC3971931

[ref50] Knutsen P. M. Ahissar E. (2009). Orthogonal coding of object location. Trends Neurosci. 32, 101–109. doi: 10.1016/j.tins.2008.10.002, PMID: 19070909

[ref51] Knutsen P. M. Derdikman D. Ahissar E. (2005). Tracking whisker and head movements in unrestrained behaving rodents. J. Neurophysiol. 93, 2294–2301. doi: 10.1152/jn.00718.2004 15563552

[ref52] Ko H. Hofer S. B. Pichler B. Buchanan K. A. Sjöström P. J. Mrsic-Flogel T. D. (2011). Functional specificity of local synaptic connections in neocortical networks. Nature 473, 87–91. doi: 10.1038/nature09880, PMID: 21478872 PMC3089591

[ref53] Komiyama T. Sato T. R. O'Connor D. H. Zhang Y. X. Huber D. Hooks B. M.. (2010). Learning-related fine-scale specificity imaged in motor cortex circuits of behaving mice. Nature 464, 1182–1186. doi: 10.1038/nature08897, PMID: 20376005

[ref54] Krupa D. J. Matell M. S. Brisben A. J. Oliveira L. M. Nicolelis M. A. (2001). Behavioral properties of the trigeminal somatosensory system in rats performing whisker-dependent tactile discriminations. J. Neurosci. 21, 5752–5763. doi: 10.1523/JNEUROSCI.21-15-05752.2001, PMID: 11466447 PMC6762640

[ref55] Kuruppath P. Gugig E. Azouz R. (2014). Microvibrissae-based texture discrimination. J. Neurosci. 34, 5115–5120. doi: 10.1523/JNEUROSCI.4217-13.2014, PMID: 24719091 PMC6608996

[ref56] Lankarany M. Al-Basha D. Ratté S. Prescott S. A. (2019). Differentially synchronized spiking enables multiplexed neural coding. Proc. Natl. Acad. Sci. 116, 10097–10102. doi: 10.1073/pnas.1812171116, PMID: 31028148 PMC6525513

[ref57] Lestienne R. (2001). Spike timing, synchronization and information processing on the sensory side of the central nervous system. Prog. Neurobiol. 65, 545–591. doi: 10.1016/S0301-0082(01)00019-3 11728644

[ref58] Lichtenstein S. H. Carvell G. E. Simons D. J. (1990). Responses of rat trigeminal ganglion neurons to movements of vibrissae in different directions. Somatosens. Mot. Res. 7, 47–65. doi: 10.3109/08990229009144697 2330787

[ref59] Lottem E. Azouz R. (2008). Dynamic translation of surface coarseness into whisker vibrations. J. Neurophysiol. 100, 2852–2865. doi: 10.1152/jn.90302.2008, PMID: 18799602

[ref60] Lottem E. Azouz R. (2009). Mechanisms of tactile information transmission through whisker vibrations. J. Neurosci. 29, 11686–11697. doi: 10.1523/JNEUROSCI.0705-09.2009, PMID: 19759315 PMC6665773

[ref61] Maldonado P. E. Friedman-Hill S. Gray C. M. (2000). Dynamics of striate cortical activity in the alert macaque: II. Fast time scale synchronization. Cereb Cortex 10, 1117–1131. doi: 10.1093/cercor/10.11.1117, PMID: 11053232

[ref62] Martini F. J. Molano-Mazon M. Maravall M. (2017). Interspersed distribution of selectivity to kinematic stimulus features in Supragranular layers of mouse barrel cortex. Cereb. Cortex 27, 3782–3789. doi: 10.1093/cercor/bhx019, PMID: 28334121

[ref63] Mehta S. B. Whitmer D. Figueroa R. Williams B. A. Kleinfeld D. (2007). Active spatial perception in the vibrissa scanning sensorimotor system. PLoS Biol. 5:e15. doi: 10.1371/journal.pbio.0050015, PMID: 17227143 PMC1769422

[ref64] Mitchinson B. Martin C. J. Grant R. A. Prescott T. J. (2007). Feedback control in active sensing: rat exploratory whisking is modulated by environmental contact. Proc. R. Soc. Lond. B Biol. Sci. 274, 1035–1041. doi: 10.1098/rspb.2006.0347 PMC2124479 17331893

[ref65] Morita T. Kang H. Wolfe J. Jadhav S. P. Feldman D. E. (2011). Psychometric curve and behavioral strategies for whisker-based texture discrimination in rats. PLoS One 6:e20437. doi: 10.1371/journal.pone.0020437, PMID: 21673811 PMC3106007

[ref66] Narayanan R. T. Udvary D. Oberlaender M. (2017). Cell type-specific structural Organization of the six Layers in rat barrel cortex. Front. Neuroanat. 11:91. doi: 10.3389/fnana.2017.00091, PMID: 29081739 PMC5645532

[ref67] O'Connor D. H. Clack N. G. Huber D. Komiyama T. Myers E. W. Svoboda K. (2010). Vibrissa-based object localization in head-fixed mice. J. Neurosci. 30, 1947–1967. doi: 10.1523/JNEUROSCI.3762-09.2010, PMID: 20130203 PMC6634009

[ref68] Oladazimi M. Brendel W. Schwarz C. (2018). Biomechanical texture coding in rat whiskers. Sci. Rep. 8:11139. doi: 10.1038/s41598-018-29225-9, PMID: 30042423 PMC6057990

[ref69] Palm G. (1990). Cell assemblies as a guidline for brain reseach. Conc Neurosci 1, 133–148.

[ref70] Peron S. P. Freeman J. Iyer V. Guo C. Svoboda K. (2015). A cellular resolution map of barrel cortex activity during tactile behavior. Neuron 86, 783–799. doi: 10.1016/j.neuron.2015.03.027, PMID: 25913859

[ref71] Pinto D. J. Brumberg J. C. Simons D. J. (2000). Circuit dynamics and coding strategies in rodent somatosensory cortex. J. Neurophysiol. 83, 1158–1166. doi: 10.1152/jn.2000.83.3.1158 10712446

[ref72] Poulet J. F. A. Petersen C. C. H. (2008). Internal brain state regulates membrane potential synchrony in barrel cortex of behaving mice. Nature 454, 881–885. doi: 10.1038/nature07150, PMID: 18633351

[ref73] Quist B. W. Hartmann M. J. (2012). Mechanical signals at the base of a rat vibrissa: the effect of intrinsic vibrissa curvature and implications for tactile exploration. J. Neurophysiol. 107, 2298–2312. doi: 10.1152/jn.00372.2011, PMID: 22298834 PMC3362248

[ref74] Reyes-Puerta V. Sun J. J. Kim S. Kilb W. Luhmann H. J. (2015). Laminar and columnar structure of sensory-evoked multineuronal spike sequences in adult rat barrel cortex in vivo. Cereb. Cortex 25, 2001–2021. doi: 10.1093/cercor/bhu007, PMID: 24518757

[ref75] Ritt J. T. Andermann M. L. Moore C. I. (2008). Embodied information processing: vibrissa mechanics and texture features shape micromotions in actively sensing rats. Neuron 57, 599–613. doi: 10.1016/j.neuron.2007.12.024, PMID: 18304488 PMC4391974

[ref76] Rothschild G. Nelken I. Mizrahi A. (2010). Functional organization and population dynamics in the mouse primary auditory cortex. Nat. Neurosci. 13, 353–360. doi: 10.1038/nn.2484, PMID: 20118927

[ref77] Sachdev R. N. S. Berg R. W. Champney G. Kleinfeld D. Ebner F. F. (2003). Unilateral vibrissa contact: changes in amplitude but not timing of rhythmic whisking. Somatosens. Mot. Res. 20, 163–169. doi: 10.1080/08990220311000405208, PMID: 12850826

[ref78] Sachdev R. N. Sellien H. Ebner F. (2001). Temporal organization of multi-whisker contact in rats. Somatosens. Mot. Res. 18, 91–100. doi: 10.1080/135578501012006192, PMID: 11534778

[ref79] Schwartz O. Pillow J. W. Rust N. C. Simoncelli E. P. (2006). Spike-triggered neural characterization. J. Vis. 6, 484–507. doi: 10.1167/6.4.13, PMID: 16889482

[ref80] Schwarz C. (2016). The slip hypothesis: tactile perception and its neuronal bases. Trends Neurosci. 39, 449–462. doi: 10.1016/j.tins.2016.04.008, PMID: 27311927

[ref81] Sharma H. Azouz R. (2022). Coexisting neuronal coding strategies in the barrel cortex. Cereb. Cortex 32, 4986–5004. doi: 10.1093/cercor/bhab527, PMID: 35149866

[ref82] Shoykhet M. Doherty D. Simons D. J. (2000). Coding of deflection velocity and amplitude by whisker primary afferent neurons: implications for higher level processing. Somatosens. Mot. Res. 17, 171–180. doi: 10.1080/08990220050020580, PMID: 10895887

[ref83] Simons D. J. (1978). Response properties of vibrissa units in rat SI somatosensory neocortex. J. Neurophysiol. 41, 798–820. doi: 10.1152/jn.1978.41.3.798, PMID: 660231

[ref84] Singer W. (1999). Neuronal synchrony: a versatile code for the definition of relations? Neuron 24, 111–125. 10.1016/s0896-6273(00)80821-1 10677026

[ref85] Sofroniew N. J. Vlasov Y. A. Hires S. A. Freeman J. Svoboda K. (2015). Neural coding in barrel cortex during whisker-guided locomotion. elife 4:e12559. doi: 10.7554/eLife.12559, PMID: 26701910 PMC4764557

[ref86] Softky W. R. Koch C. (1993). The highly irregular firing of cortical cells is inconsistent with temporal integration of random EPSPs. J. Neurosci. 13, 334–350. doi: 10.1523/JNEUROSCI.13-01-00334.1993, PMID: 8423479 PMC6576320

[ref87] Stuttgen M. C. Schwarz C. (2008). Psychophysical and neurometric detection performance under stimulus uncertainty. Nat. Neurosci. 11, 1091–1099. doi: 10.1038/nn.2162 19160508

[ref88] Stuttgen M. C. Schwarz C. (2010). Integration of vibrotactile signals for whisker-related perception in rats is governed by short time constants: comparison of neurometric and psychometric detection performance. J. Neurosci. 30, 2060–2069. doi: 10.1523/JNEUROSCI.3943-09.2010, PMID: 20147534 PMC6634023

[ref89] Szwed M. Bagdasarian K. Ahissar E. (2003). Encoding of vibrissal active touch. Neuron 40, 621–630. doi: 10.1016/S0896-6273(03)00671-8, PMID: 14642284

[ref90] Temereanca S. Brown E. N. Simons D. J. (2008). Rapid changes in thalamic firing synchrony during repetitive whisker stimulation. J. Neurosci. 28, 11153–11164. doi: 10.1523/JNEUROSCI.1586-08.2008, PMID: 18971458 PMC2617717

[ref91] Towal R. B. Hartmann M. J. (2006). Right–left asymmetries in the whisking behavior of rats anticipate head movements. J. Neurosci. 26, 8838–8846. doi: 10.1523/JNEUROSCI.0581-06.2006, PMID: 16928873 PMC6674387

[ref92] Towal R. B. Hartmann M. J. Z. (2008). Variability in velocity profiles during free-air whisking behavior of unrestrained rats. J. Neurophysiol. 100, 740–752. doi: 10.1152/jn.01295.2007, PMID: 18436634

[ref93] Towal R. B. Quist B. W. Gopal V. Solomon J. H. Hartmann M. J. (2011). The morphology of the rat vibrissal array: a model for quantifying spatiotemporal patterns of whisker-object contact. PLoS Comput. Biol. 7:e1001120. doi: 10.1371/journal.pcbi.1001120, PMID: 21490724 PMC3072363

[ref94] Voigts J. Herman D. H. Celikel T. (2015). Tactile object localization by anticipatory whisker motion. J. Neurophysiol. 113, 620–632. doi: 10.1152/jn.00241.2014, PMID: 25339711

[ref95] von Heimendahl M. Itskov P. M. Arabzadeh E. Diamond M. E. (2007). Neuronal activity in rat barrel cortex underlying texture discrimination. PLoS Biol. 5:e305. doi: 10.1371/journal.pbio.0050305, PMID: 18001152 PMC2071938

[ref96] Waiblinger C. Brugger D. Schwarz C. (2013). Vibrotactile discrimination in the rat whisker system is based on neuronal coding of instantaneous kinematic cues. Cereb. Cortex 25, 1093–1106. doi: 10.1093/cercor/bht305 24169940 PMC4380004

[ref97] Waiblinger C. Brugger D. Whitmire C. J. Stanley G. B. Schwarz C. (2015). Support for the slip hypothesis from whisker-related tactile perception of rats in a noisy environment. Front. Integr. Neurosci. 9:53. doi: 10.3389/fnint.2015.00053 26528148 PMC4606012

[ref98] Welker W. I. (1964). Analysis of sniffing of the albino rat 1. Behaviour 22, 223–244. doi: 10.1163/156853964X00030

[ref99] Welker C. Woolsey T. A. (1974). Structure of layer IV in the somatosensory neocortex of the rat: description and comparison with the mouse. J. Comp. Neurol. 158, 437–453. doi: 10.1002/cne.901580405 4141363

[ref100] Wolfe J. Hill D. N. Pahlavan S. Drew P. J. Kleinfeld D. Feldman D. E. (2008). Texture coding in the rat whisker system: slip-stick versus differential resonance. PLoS Biol. 6:e215. doi: 10.1371/journal.pbio.0060215, PMID: 18752354 PMC2525689

[ref101] Womelsdorf T. Fries P. (2007). The role of neuronal synchronization in selective attention. Curr. Opin. Neurobiol. 17, 154–160. doi: 10.1016/j.conb.2007.02.002, PMID: 17306527

[ref102] Woolsey T. A. Van der Loos H. (1970). The structural organization of layer IV in the somatosensory region (SI) of mouse cerebral cortex. The description of a cortical field composed of discrete cytoarchitectonic units. Brain Res. 17, 205–242. doi: 10.1016/0006-8993(70)90079-X 4904874

[ref103] Zhang M. Alloway K. D. (2004). Stimulus-induced intercolumnar synchronization of neuronal activity in rat barrel cortex: a laminar analysis. J. Neurophysiol. 92, 1464–1478. doi: 10.1152/jn.01272.2003, PMID: 15056676

[ref104] Zhang M. Alloway K. D. (2006). Intercolumnar synchronization of neuronal activity in rat barrel cortex during patterned airjet stimulation: a laminar analysis. Exp. Brain Res. 169, 311–325. doi: 10.1007/s00221-005-0152-5, PMID: 16284753

[ref105] Zuo Y. Diamond M. E. (2019). Rats generate Vibrissal sensory evidence until boundary crossing triggers a decision. Curr. Biol. 29, 1415–1424.e5. doi: 10.1016/j.cub.2019.03.016, PMID: 31006570

[ref106] Zuo Y. Perkon I. Diamond M. E. (2011). Whisking and whisker kinematics during a texture classification task. Philos. Trans. R. Soc. Lond. Ser. B Biol. Sci. 366, 3058–3069. doi: 10.1098/rstb.2011.0161, PMID: 21969687 PMC3172602

