# Supplementary material for: Global and local neuronal coding of tactile information in the barrel cortex
Source: Front Neurosci. 2024 Jan 5;17:1291864. doi: 10.3389/fnins.2023.1291864 (PMC10796699; doi:10.3389/fnins.2023.1291864)
Supplement: Supplementary file 1 [file Data_Sheet_1.PDF]

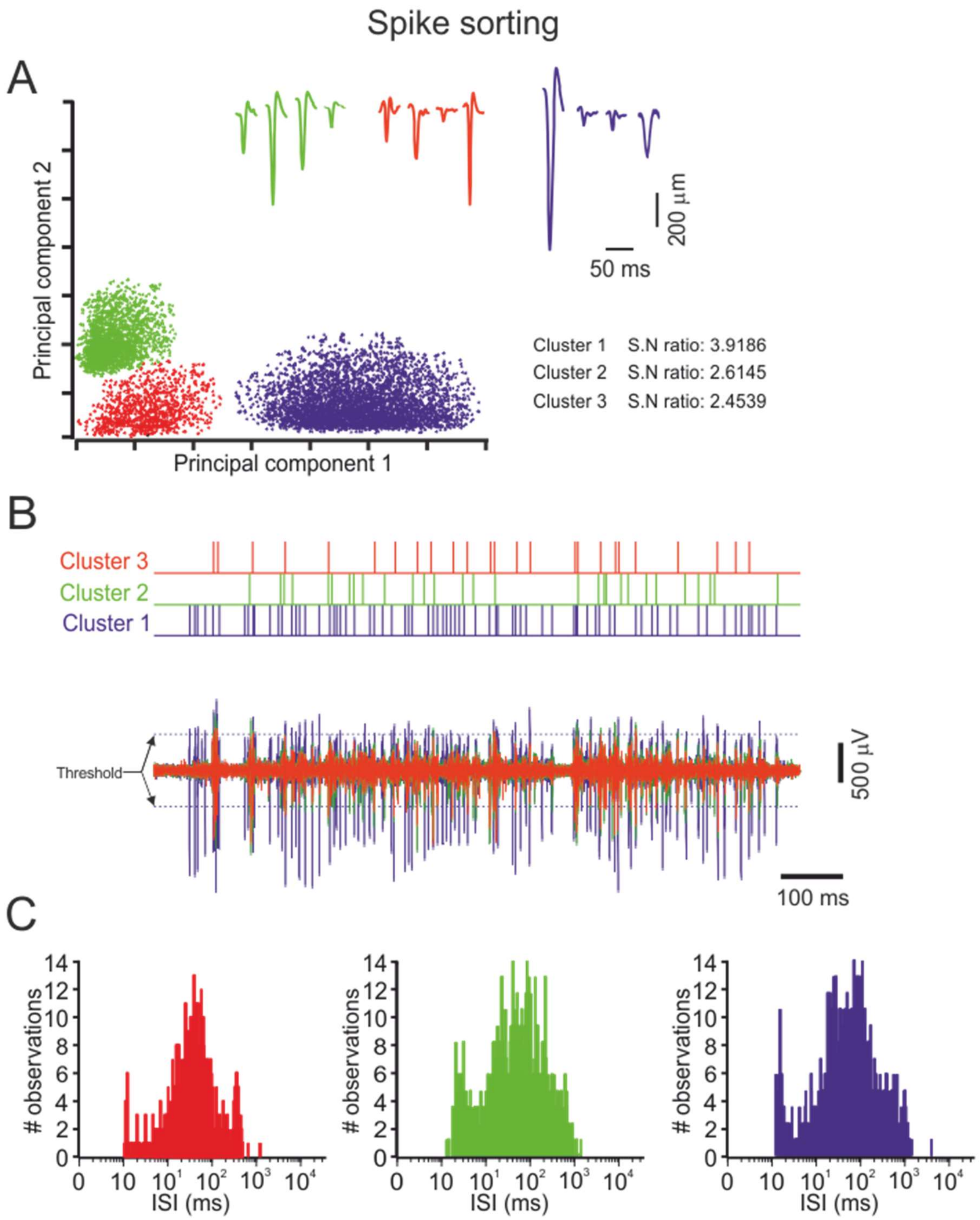

Supplementary Figure S1. Spike sorting. (**A**). Scatterplots of spikes in feature space, color-coded by putative identity. Spike waveforms recorded on a single tetrode were projected onto a 2D feature space. Scatterplot of the first and second principal components from channels 1 and 4, respectively. The inset shows the spike waveforms for the three units in the scatter plot. (**B**). Example of raw data and the results of spike sorting as color-coded traces (bottom) and after digitization (top). (**C**). ISIH of the three units.

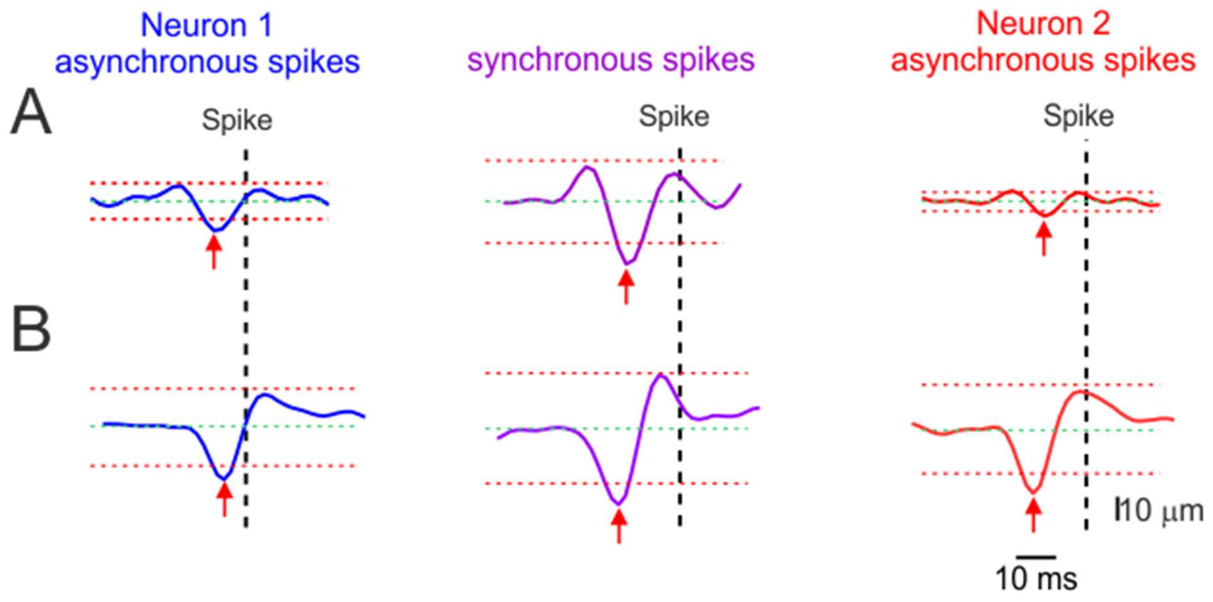

Supplementary Figure S2. Examples of SSE for synchronous and asynchronous spikes from two different sessions. The dashed vertical lines indicate spikes. (**A**). Spike latency from its underlying SSE (red arrow to vertical dashed line) for synchronous (middle panels) and asynchronous spikes (left and right panels) the dashed green and red horizontal lines indicate Mean±3SD of each trace, respectively.
